# The impact of long-term exercise on liver function, fatty liver progression, and related metabolic markers in NAFLD patients: a meta-analysis of randomized controlled trials

**DOI:** 10.3389/fnut.2026.1731510

**Published:** 2026-03-30

**Authors:** Yongqing Guo, Xianyang Xin, Pei Liu

**Affiliations:** Capital University of Physical Education and Sports, Beijing, China

**Keywords:** liver function, meta-analysis, metabolism, non-alcoholic fatty liver disease, exercise

## Abstract

**Background:**

Evidence on the effects of long-term exercise on liver function, fatty liver progression, and metabolic markers in non-alcoholic fatty liver disease (NAFLD) patients is limited.

**Objective:**

This study aimed to assess, through a meta-analysis, the impact of long-term exercise on liver function, fatty liver progression, and related metabolic indicators in NAFLD.

**Methods:**

The study investigated 5 databases and included 18 studies published up to September 2025. A random-effects model was used, with subgroup and regression analyses to explore intervention effects.

**Results:**

Long-term exercise significantly reduced alanine aminotransferase (ALT; standardized mean differences, SMD = −0.78, 95% CI: −1.15 to −0.40), aspartate aminotransferase (AST; SMD = −0.65, 95% CI: −1.07 to −0.23), and liver fat content (SMD = −0.68, 95% CI: −0.98 to −0.38). Additionally, significant improvements were observed in body weight, body mass index (BMI), visceral adipose tissue (VAT), and homeostasis model assessment of insulin resistance (HOMA-IR). No significant changes in liver stiffness and glycated hemoglobin percentage (HbA1c%) were observed, which may be due to the limited number of studies on liver stiffness (only three studies were included). Subgroup analyses identified exercise type, gender, and region as moderators of the intervention effects. Regression analysis showed that BMI was significantly correlated with age (*p* = 0.02), while no other variables showed significant correlations.

**Conclusion:**

Long-term exercise significantly improves liver enzymes (ALT and AST) and fat content in NAFLD patients, together with BMI, VAT, and HOMA-IR, but has no significant effects on liver stiffness and HbA1c%. Nevertheless, the statistical power of this meta-analysis is limited due to the small sample sizes of the included studies and the restricted number of studies available for certain outcomes, with liver stiffness evaluated in only four studies.

**Systematic review registration:**

https://www.crd.york.ac.uk/, Identifier CRD420251163167.

## 1 Introduction

Non-alcoholic fatty liver disease (NAFLD) is the most common liver disease worldwide and has become an important risk factor for liver cirrhosis, liver cancer, and cardiovascular diseases (1, 2). Among all common cancers in the United States, hepatocellular carcinoma (HCC) is the only tumor with an increasing mortality rate (3). The prevalence of advanced liver disease and the demand for liver transplants are rising (4). Despite some promising advancements, there are currently no FDA-approved drug therapies (5). As a result, lifestyle interventions, particularly exercise, have gained increasing attention as a potential therapeutic strategy for NAFLD patients (6).

Extant research has demonstrated that exercise can attenuate NAFLD by improving insulin sensitivity, reducing hepatic fat accumulation, and mitigating systemic inflammation (7, 8). However, despite several small-scale randomized controlled trials (RCTs) that explore the effects of long-term exercise on liver function, fatty liver progression, and associated metabolic parameters in NAFLD patients, the overall body of evidence remains insufficient, with notable heterogeneity across studies (9). A considerable number of existing meta-analyses incorporate not only RCTs but also non-randomized controlled trials (non-RCTs), including cohort and observational studies, which may compromise the validity and comparability of the results. For instance, the meta-analysis by Houttu et al. (10), included six non-RCT studies, while Golabi et al. (11) incorporated cohort studies. The inclusion of non-RCTs may introduce bias and affect the overall reliability of conclusions. Meanwhile, studies such as those by Smart et al. (12), included obese and overweight healthy adults, while Wang’s (13) study included patients with additional metabolic diseases, without clearly defining the diagnostic criteria for NAFLD. These mixed populations increased heterogeneity and raised concerns about the specificity of exercise interventions for NAFLD. Additionally, most existing meta-analyses have predominantly focused on short-term interventions or single exercise modalities (14). Rahimi (14) evaluated only the effect of aerobic training (AT) on NAFLD patients, and Ghaffari (15) assessed only the effect of AT on NAFLD metabolic parameters. These studies failed to comprehensively analyze the effects of various exercise types, intervention durations, and frequencies on NAFLD. Furthermore, most studies have not sufficiently accounted for individual differences such as gender, age, and weight, which could moderate the intervention outcomes (16–18).

To address these issues, this study will include RCTs that meet strict inclusion criteria and conduct a systematic review and meta-analysis to comprehensively assess the effects of long-term exercise interventions on liver function, fatty liver progression, and related metabolic parameters in NAFLD patients, while comparing the effects of different exercise modalities. The primary outcome measures will include liver function indicators (such as ALT, AST, liver fat content, and liver stiffness) and metabolic parameters (such as BMI, weight, visceral fat, HOMA-IR, and glycated hemoglobin percentage; HbA1c%). Additionally, this study will use a random-effects model for the meta-analysis and will explore the impact of exercise type and participant demographics on the results through subgroup analysis. Regression analysis will also be used to assess the moderating effects of individual differences, including gender, age, and body composition, on intervention outcomes.

## 2 Methods

This study follows the reporting guidelines for meta-analyses (PRISMA) (19) and is conducted according to the methodological guidance of the Cochrane Handbook for Systematic Reviews of Interventions (20). It has been registered in the PROSPERO database (Registration No.: CRD420251163167), and the protocol is available for access through the PROSPERO database without any modifications to the registered or protocol information.

### 2.1 Literature search strategy

This study will systematically search the PubMed, Cochrane Library, Web of Science, Elton B. Stephens Company (EBSCO), and Embase databases to collect RCTs on the effects of exercise on liver function, fatty liver progression, and related metabolic parameters in populations with NAFLD. The core search strategy framework will include terms such as “exercise AND non-alcoholic fatty liver disease AND randomized controlled trials,” with additional expansions using related keywords and Boolean operators to ensure comprehensive coverage. This study will include research published from the establishment of each database up to September 2025, excluding studies with issues related to data consistency, methodology, or other relevant factors. Additionally, references to previously published reviews and meta-analyses will be manually screened to supplement any potentially missed studies. The specific search strategies for each database are provided in the Supplementary Tables A1–A5.

### 2.2 Inclusion and exclusion criteria

#### 2.2.1 Inclusion criteria

The criteria were developed according to the Participants, Intervention, Comparison, Outcome, and Study Design (PICOS) framework. The inclusion criteria are as follows:

1. Adults (≥18 years old) who are overweight or obese and have been diagnosed with NAFLD;
2. Exercise interventions included in this study are AT, resistance training (RT), and high-intensity interval training (HIIT), including but not limited to various forms of exercise;
3. No restrictions apply to indoor or outdoor settings, supervised or unsupervised methods;
4. In this study, “long-term exercise” is defined as interventions lasting at least 8 weeks. Only long-term exercise interventions with a duration of at least 8 weeks are included.
5. The study design must be an RCT with a clear intervention group and a control group, where the control group receives no exercise intervention or only receives advice on routine physical activity without an actual exercise load;
6. The study reports data on liver function or fatty liver progression or certain related metabolic parameters before and after intervention, as primary and secondary outcome measures. Primary outcome measures include serum ALT, AST, liver fat content, and liver stiffness. Secondary outcome measures include BMI, weight, VAT, HOMA-IR, and HbA1c%.

#### 2.2.2 Exclusion criteria

1. Non-English language articles;
2. Conference abstracts, theses, short reports, or studies where full-text access is unavailable;
3. Studies where outcome data cannot be extracted and cannot be obtained by contacting the authors;
4. Studies involving adolescents or children (<18 years old);
5. Studies involving patients with severe liver diseases such as cirrhosis, NASH (defined as ≥5% liver fat accumulation accompanied by hepatocellular injury and inflammation) (21), or liver cancer;
6. Studies combining exercise with dietary interventions to ensure the intervention factor is solely exercise;
7. Studies without a control group;
8. Studies including observational, cohort, or case–control studies.

### 2.3 Literature screening and data extraction

Two researchers (YG and XX) independently completed the literature screening, data extraction, and also conducted cross-checking. Any disagreements were resolved with assistance from a third party (PL). Missing data were supplemented by contacting the original authors. During the literature screening, titles and abstracts were first reviewed to exclude clearly unrelated studies, and the full text was then read to confirm eligibility for inclusion.

Data extraction included primary outcomes (ALT, AST, liver fat content, and liver stiffness) and secondary outcomes (BMI, weight, visceral fat, HOMA-IR, and HbA1c%), as well as information on outcome measures, sample size, and intervention details. For missing data, efforts were made to contact the original authors for supplementation. If no response was received within 3 weeks, the study was excluded.

### 2.4 Risk of bias assessment for included studies

Two researchers (YG and PL) independently assessed the risk of bias for each study using the RCT risk of bias (ROB1) tool from the Cochrane Handbook (18). According to the criteria (19), if there were no high-risk entries and the number of unclear risk entries was ≤3, the study was classified as high quality; if there was one high-risk entry and the number of unclear risk entries was ≥4, the study was classified as medium quality; all other cases were classified as low quality. The overall evaluation is presented in the Supplementary Table A5.

### 2.5 Other analyses

To explore the sources of heterogeneity and moderating factors between studies, this study conducted subgroup analysis and meta-regression analysis. Subgroup analysis explored heterogeneity sources from three dimensions: intervention type, gender, and study region. Meta-regression analysis considered variables such as intervention duration, weekly intervention frequency, session duration, and age.

### 2.6 Statistical methods

Data analysis was performed using the *meta* and *metafor* packages in R software (version 4.5.1) (22). To reduce the impact of baseline differences, this study combined the mean differences (MDs) and their standard deviation both before and after the interventions, to calculate the effect size. Considering the inconsistency of units across studies, a random-effects model will be used for the meta-analysis to accurately estimate the overall effect. All results are presented using forest plots, showing the effect size and its 95% confidence interval. BMI and weight will be analyzed using MDs, while other indicators are analyzed using standardized mean differences (SMDs) and their 95% confidence intervals. *I^2^* is used to quantify the degree of statistical heterogeneity. Subgroup analysis results will be presented in tables, regression analysis results in regression plots, and funnel plots will be used to assess publication bias. Quantitative and qualitative analyses of publication bias will be performed using the Begg’s test and Egger’s regression test. If the *p* < 0.05, the trim-and-fill method will be used to adjust for bias. Additionally, sensitivity analysis will be conducted using the leave-one-out method to assess the stability of the results.

The first step involved calculating the difference in means:


$$M_{\text{change}}=M_{\text{post}}-M_{\mathrm{pre}}$$

where *M_change_* is the raw mean difference, *M_post_* is the reported mean post-intervention, and *M_pre_* is the reported mean pre-intervention (23). Then, the standard deviation (SD) of the change in means is calculated as follows (23):


$$SD_{\text{change}}=\sqrt{SD_{\mathrm{pre}}^{2}+SD_{\text{post}}^{2}-(2\times r\times SD_{\mathrm{pre}}\times SD_{\text{post}})}$$

where *SD_change_* is the standard deviation of the difference in means, *SD_pre_* is the SD from pre-intervention, *SD_post_* is the SD from post-intervention, and *r* is the correlation coefficient (23). Correlation coefficients for pre- and post-intervention were rarely reported in the included studies and were generally assumed to be *r* = 0.50, as suggested by the Cochrane Handbook (23).

## 3 Results

### 3.1 Literature screening process

**Figure 1:**
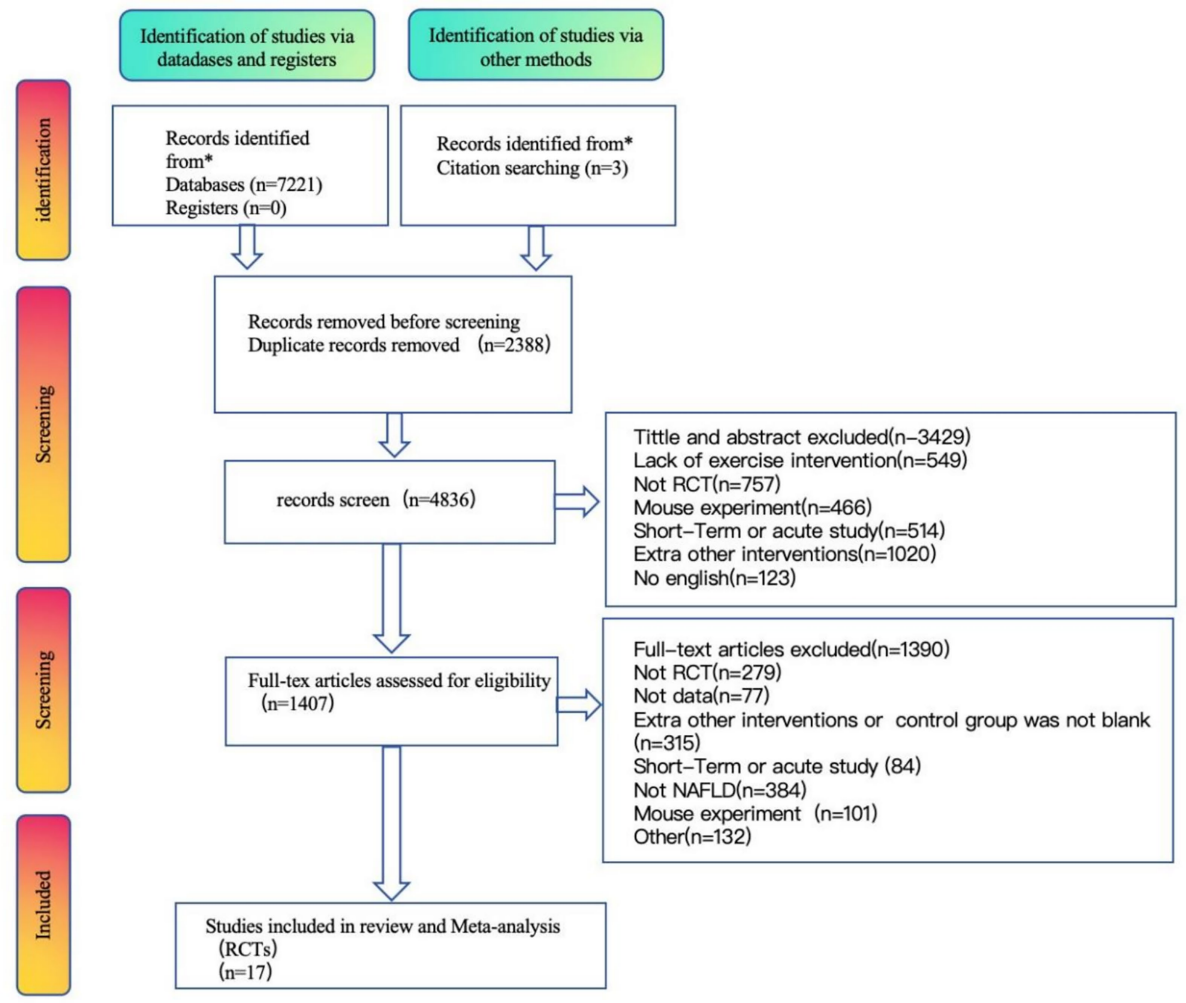
Flow chart of the study’s selection process.

A total of 7,221 relevant articles were obtained through database searches, including PubMed (469), Embase (4,005), Cochrane Library (655), EBSCO (550), and Web of Science (1,542), with an additional 3 articles obtained from other sources. Using EndNote 20 software, 2,388 duplicate articles were removed. Two researchers independently screened the titles and abstracts, excluding 3,429 articles, and then conducted full-text reviews, excluding 1,390 articles. Ultimately, 17 articles were included. The literature selection process is shown in Figure 1.

### 3.2 Basic characteristics of the included studies

A total of 754 participants were included in the studies. The participants’ age ranged from 35 to 63 years, and their BMI ranged from 27.8 to 38.1 kg/m^2^. The most common intervention was AT (12 studies), followed by RT (5 studies) and HIIT (4 studies). The exercise intervention frequency ranged from 2 to 5 times per week, with three times per week being the most common. Detailed basic characteristics of the included studies are presented in Table 1.

**Table 1:** Basic characteristics of included studies.

Author	Region	Sample measure				Period (week)	Frequency (times/week)	Minutes	Outcome
		Exp	Con	Exp	Con				
Abdelbasset et al. (31)	Egypt	15	8	AT	Conventional treatment	8	3	13–14	①③④⑥⑦⑧
Abdelbasset et al. (31)	Egypt	16	8	HIIT	Conventional treatment	8	3	40–50	①③④⑥⑦⑧
Ezpeleta et al. (58)	United States	15	20	AT	No intervention	12	5	60	①②③④⑤⑥⑦⑧
Guo et al. (59)	Iran	20	10	AT	Lifestyle guidance	12	3	45–60	①②③④⑤⑥⑧⑨
Guo et al. (59)	Iran	20	10	HIIT	Lifestyle guidance	12	3	45–60	①②③④⑤⑥⑧⑨
Naimimohasses et al.(60)	Ireland	16	14	AT	Standard treatment	12	2	31–56	①⑧⑨
Cheng et al. (32)	United States	22	18	AT	No intervention	24	2–3	30–60	②④⑤⑦
Zelber-Sagi et al. (61)	Israel	33	31	RT	Stretching exercises	12	3	40	①②④⑤⑥⑦⑧
Hui-Jie Zhang et al. (62)	China	73	37	AT	No intervention	52	5	30	②③④⑤
Hui-Jie Zhang et al. (62)	China	73	37	AT	No intervention	52	5	30	②③④⑤
Csader et al. (63)	Finland	7	7	HIIT	No intervention	12	3	45–60	①③④⑤⑦⑧
Hallsworth et al. (64)	United Kingdom	11	12	HIIT	Standard treatment	12	3	30–40	①②③④⑤⑥⑦⑧
Rezende et al. (65)	Brazil	19	21	AT	No intervention	24	2	40–60	①④⑤⑥⑦
Hoseini et al. (66)	Iran	10	10	AT	Placebo	8	5	45–60	①②⑥
Cuthbertson et al. (67)	United Kingdom	30	20	AT	Regular consultation	12	3	30	①②④⑤⑥
Hallsworth et al. (68)	United Kingdom	11	8	RT	Conventional treatment	8	3	45–60	⑥⑧
Shamsoddini et al. (8)	Iran	10	5	AT	No intervention	8	3	45	①②④⑤
Shamsoddini et al. (8)	Iran	10	5	RT	No intervention	8	3	45	①②④⑤
Mohammadi et al. (69)	Iran	10	10	RT	No intervention	12	3	45–60	①④⑤
Moradi Kelardeh et al. (70)	Iran	12	12	RT	Placebo	12	3	45–60	①④⑤
Sullivan et al. (71)	United States	12	6	AT	Observation	16	5	30–60	②④

①, BMI; ②, Weight; ③, visceral adipose tissue (VAT); ④, alanine aminotransferase (ALT); ⑤, aspartate aminotransferase (AST); ⑥, homeostasis model assessment of insulin resistance (HOMA-IR); ⑦, glycated hemoglobin percentage (HbA1c%); ⑧, liver fat; and ⑨, liver stiffness.

### 3.3 Basic characteristics and risk of bias assessment results of included studies

This meta-analysis included 17 articles. Among the 17 included studies, 16 clearly described the allocation method, 1 reported allocation concealment, and 4 reported blinding of outcome assessment. Regarding data completeness, four studies had a dropout rate exceeding 20% and did not use intention-to-treat analysis, which was rated as high risk. Further, eight studies reported dropout rates, with similar dropout numbers and reasons between the intervention and control groups. Other sources of bias included small sample sizes (fewer than 10 participants), lack of supervision in exercise interventions, and conflicts of interest, and 14 studies did not report other sources of bias. The results of the risk of bias assessment are shown in the Supplementary material, Figure 2 and Supplementary Figure A1.

**Figure 2:**
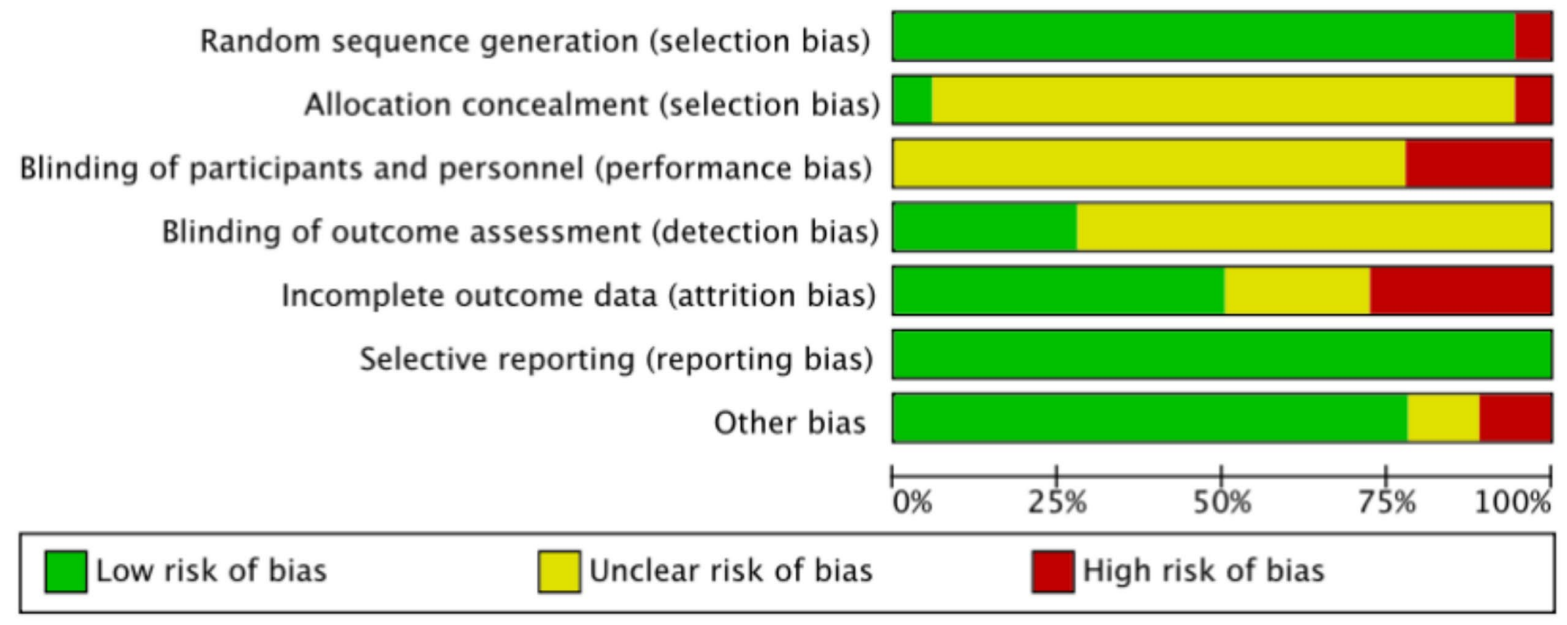
Overview of risk of bias assessment results.

### 3.4 Meta-analysis results

#### 3.4.1 Primary outcome measures

**Figure 3:**
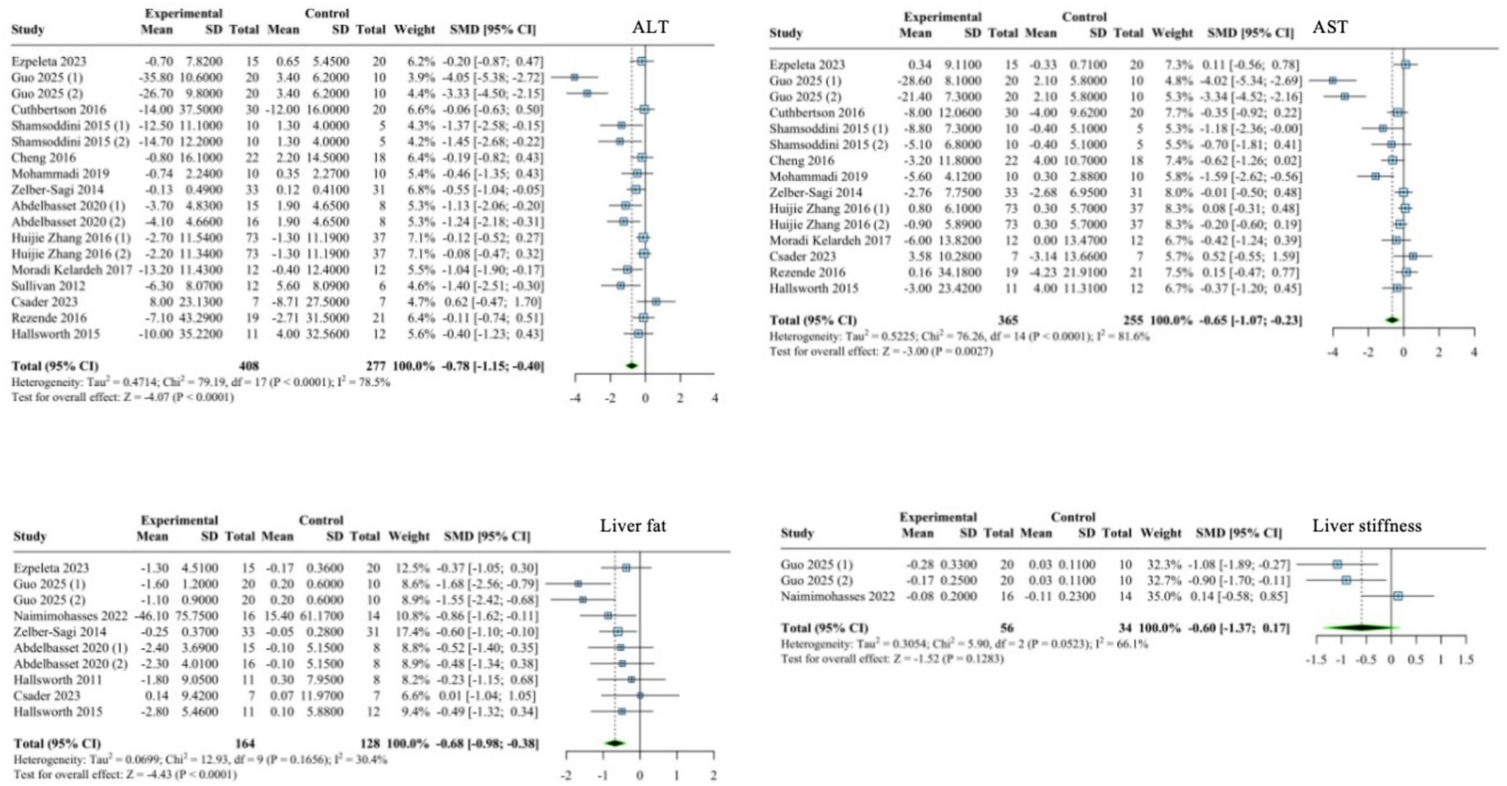
Forest plot of the effect of exercise on liver function and the progression of fatty liver indicators.

The random-effects model analysis showed that the effect of exercise on ALT was SMD = −0.78 (95% CI: −1.15 to −0.40), *p* < 0.001; the heterogeneity test results showed *I^2^* = 78.5%, *p* < 0.001. The effect of exercise on AST was SMD = −0.65 (95% CI: −1.07 to −0.23), *p* = 0.003; and the heterogeneity test results showed *I^2^* = 81.6%, *p* < 0.001. The effect of exercise on liver fat was SMD = −0.68 (95% CI: −0.98 to −0.38), *p* < 0.0001; and the heterogeneity test result showed *I^2^* = 30.4%, *p =* 0.166. The effect of exercise on liver stiffness was SMD = −0.60 (95% CI: −1.37 to 0.17), *p* = 0.128; and the heterogeneity test results showed *I^2^* = 66.1%, *p* = 0.052. Details are shown in Figure 3.

#### 3.4.2 Secondary outcome measures

**Figure 4:**
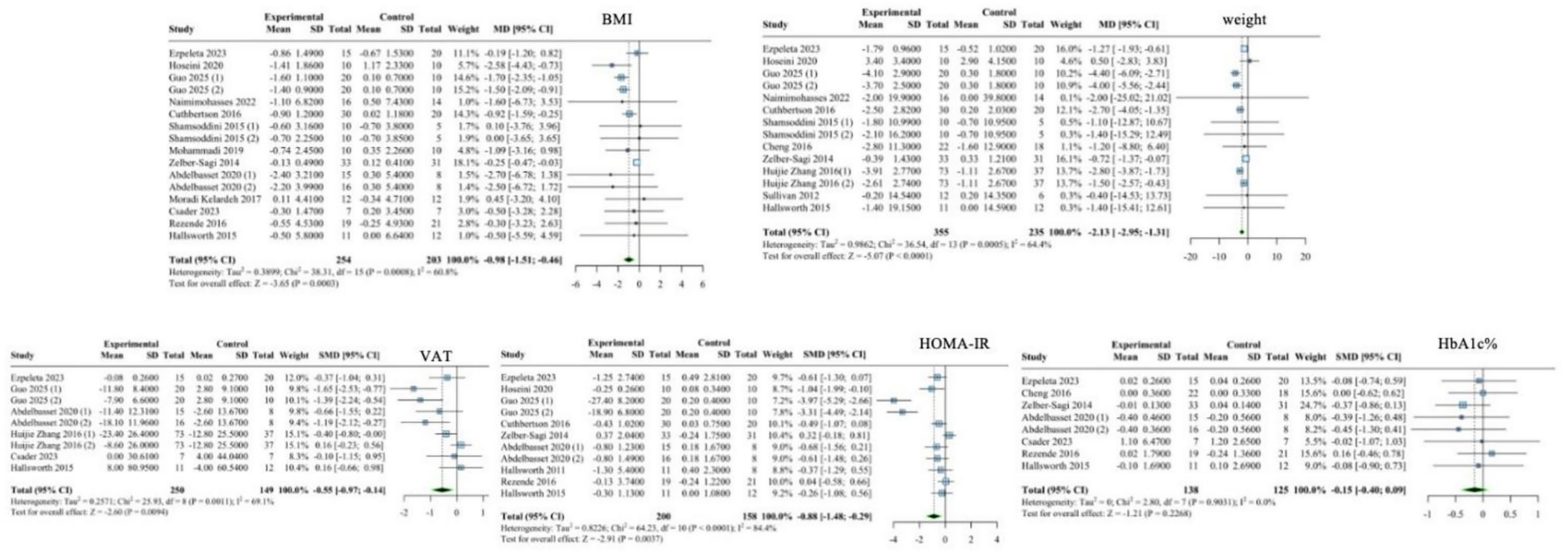
Forest plot of the effect of exercise on related metabolic markers.

The random-effects model analysis showed that the effect of exercise on BMI was MD = −0.98 (95% CI: −1.51 to −0.46), *p* < 0.001; the heterogeneity test results showed *I*^2^ = 60.8%, *p* < 0.001. The effect of exercise on weight was MD = −2.13 (95% CI: −2.95 to −1.31), *p* < 0.001; and the heterogeneity test results showed *I*^2^ = 64.4%, *p* < 0.001. The effect of exercise on VAT was SMD = −0.55 (95% CI: −0.97 to −0.14), *p* = 0.009; the heterogeneity test results showed *I*^2^ = 69.1.4%, *p* = 0.001. The effect of exercise on HOMA-IR was SMD = −0.88 (95% CI: −1.48 to −0.29), *p* = 0.004; and the heterogeneity test results showed *I*^2^ = 84.4%, *p* < 0.001. The effect of exercise on HbA1c% was SMD = −0.15 (95% CI: −0.40 to 0.09), *p* = 0.227; and the heterogeneity test results showed *I*^2^ = 0%, *p* = 0.903. Details are shown in Figure 4.

### 3.5 Subgroup analysis results

Subgroup analyses were performed for all outcome measures. The categorization into subgroups based on gender, exercise type, and continent was made according to prior research that highlighted the potential influence of these factors on the effects of exercise on NAFLD (24). In addition, considering that diabetes is a common comorbidity of NAFLD, it may significantly impact on liver metabolic function and the effectiveness of exercise interventions. Therefore, this study also included patients with comorbid diabetes in the subgroup analysis. Most outcome measures did not show significant differences in the subgroup analyses based on gender, exercise type, continent, and diabetes. However, significant differences in BMI and weight were observed in the exercise type subgroup (*p* < 0.001 and *p* < 0.001, respectively). Specifically, HIIT showed the most pronounced improvement, while RT had the least beneficial effect on these parameters. Regarding liver stiffness, significant differences were observed across continents (*p* = 0.016). More specifically, Asians showed the most significant improvement in liver stiffness. Regarding diabetes comorbidity, BMI reduction was more pronounced in patients without comorbid diabetes (*p* < 0.001). While significant differences were also observed in other diabetes subgroups, these differences were classified as “unclear” because the inclusion criteria of this study encompassed diabetic patients, but it remains uncertain whether they were ultimately included in the analysis. No significant differences were found for other measures between the groups (see details in Table 2 and Supplementary Table A7).

**Table 2:** Subgroup analysis table for liver function and the progression of fatty liver indicators.

Moderating variables	Subgroup analysis	K	MD/SMD (95% CI)	*I* ^2^	*p*
ALT					
Type	AT	10	−0.6575 [−1.1214 to −0.1937]	79.3	
	RT	4	−0.7042 [−1.0750 to −0.3334]	0	
	HIIT	4	−1.0660 [−2.5197 to 0.3876]	88.4	
					0.871
Continent	Africa	2	−1.1875 [−1.8453 to −0.5297]	0	
	North America	3	−0.4553 [−1.0740 to 0.1635]	48.8	
	South America	1	−0.1146 [−0.7357 to 0.5065]	–	
	Asia	9	−1.2229 [−1.8887 to −0.5570]	87.3	
	Europe	3	−0.0433 [−0.4935 to 0.4069]	6.5	
					0.007
Gender	Mix	11	−0.9357 [−1.4540 to −0.4174]	85.3	
	Male	5	−0.7444 [−1.2776 to −0.2113]	40.8	
	Female	1	0.6155 [−0.4654 to 1.6964]	–	
	No clear	1	−0.3986 [−1.2263 to 0.4292]	–	
					0.075
Diabetes mellitus	Yes	6	−0.3619 [−0.8178 to 0.0940]	51.4	
	No	7	−0.4551 [−0.8168 to −0.0934]	56.2	
	No clear	5	−1.7834 [−3.1060 to −0.4608]	88.9	
					0.135
AST					
Type	AT	8	−0.5409 [−1.0647 to −0.0172]	82.6	
	RT	4	−0.5781 [−1.2402 to 0.0840]	61.4	
	HIIT	3	−1.0402 [−3.1028 to 1.0224]	91.9	
					0.900
Continent	North America	3	−0.2639 [−0.9823 to 0.4545]	58.5	
	South America	1	0.1516 [−0.4700 to 0.7731]	–	
	Asia	9	−1.1123 [−1.8016 to −0.4230]	88.3	
	Europe	3	−0.2076 [−0.6634 to 0.2482]	7.9	
					0.057
Gender	Mix	8	−0.8216 [−1.4506 to −0.1926]	92.2	
	Male	5	−0.6557 [−1.3019 to −0.0095]	64.2	
	Female	1	0.5203 [−0.5513 to 1.5918]	–	
	No clear	1	−0.3724 [−1.1989 to 0.4541]	–	
					0.187
Diabetes mellitus	Yes	4	−0.0309 [−0.4849 to 0.4231]	37.5	
	No	6	−0.1737 [−0.4214 to 0.0739]	17.6	
	No clear	5	−1.8816 [−3.2436 to −0.5196]	89.2	
					**0.041**
Liver fat					
Type	AT	4	−0.8239 [−1.3686 to −0.2791]	47.4	
	RT	2	−0.5147 [−0.9546 to −0.0748]	0	
	HIIT	4	−0.6582 [−1.2881 to −0.0283]	49.3	
					0.686
Continent	Africa	2	−0.5026 [−1.1162 to 0.1109]	0	
	North America	1	−0.3742 [−1.0501 to 0.3016]	0	
	Asia	3	−1.2014 [−1.9571 to −0.4457]	67.9	
	Europe	4	−0.4708 [−0.9049 to −0.0367]	0	
					0.355
Gender	Mix	7	−0.8155 [−1.1762 to −0.4548]	38.1	
	Female	1	0.0061 [−1.0416 to 1.0537]	0	
	No clear	2	−0.3744 [−0.9901 to 0.2413]	–	
					0.214
Diabetes mellitus	Yes	5	−0.4878 [−0.8526 to −0.1230]	0	
	No	1	−0.5995 [−1.1013 to −0.0978]	–	
	No clear	4	−0.9890 [−1.6991 to −0.2789]	62.1	
					0.469
Liver stiffness					
Type	AT	2	−0.4571 [−1.6498 to 0.7357]	86.7	
	HIIT	1	−0.9043 [−1.7018 to −0.1068]	–	
					0.541
Continent	Asia	2	−0.9911 [−1.5606 to −0.4216]	0	
	Europe	1	0.1361 [−0.5821 to 0.8543]	–	
					**0.016**
Gender	Mix	3	−0.5969 [−1.3662 to 0.1724]	66.1	
					–
Diabetes mellitus	No clear	2	−0.9911 [−1.5606 to −0.4216]	0	
	Yes	1	0.1361 [−0.5821 to 0.8543]	–	
					**0.016**

Bold values represent values greater than 0.05.

### 3.6 Meta-regression analysis results

**Figure 5:**
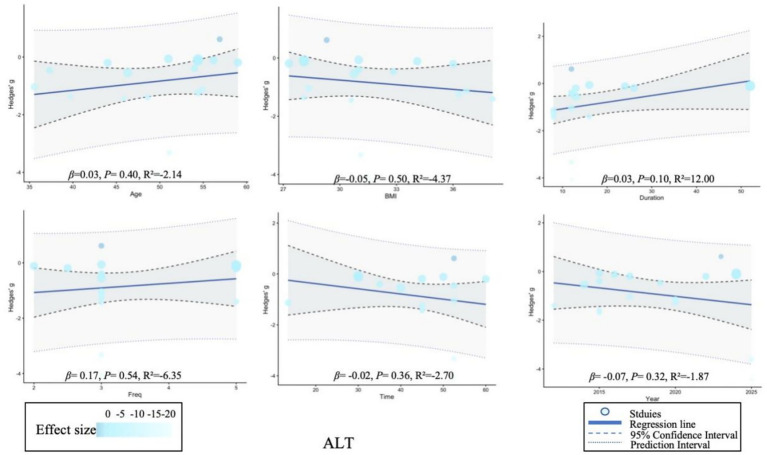
Meta-regression of the effect of exercise on alanine aminotransferase. *β* (slope), regression coefficient (slope); *p*, *p*-value for statistical significance; *R*^2^, coefficient of determination (model fit).

**Figure 6:**
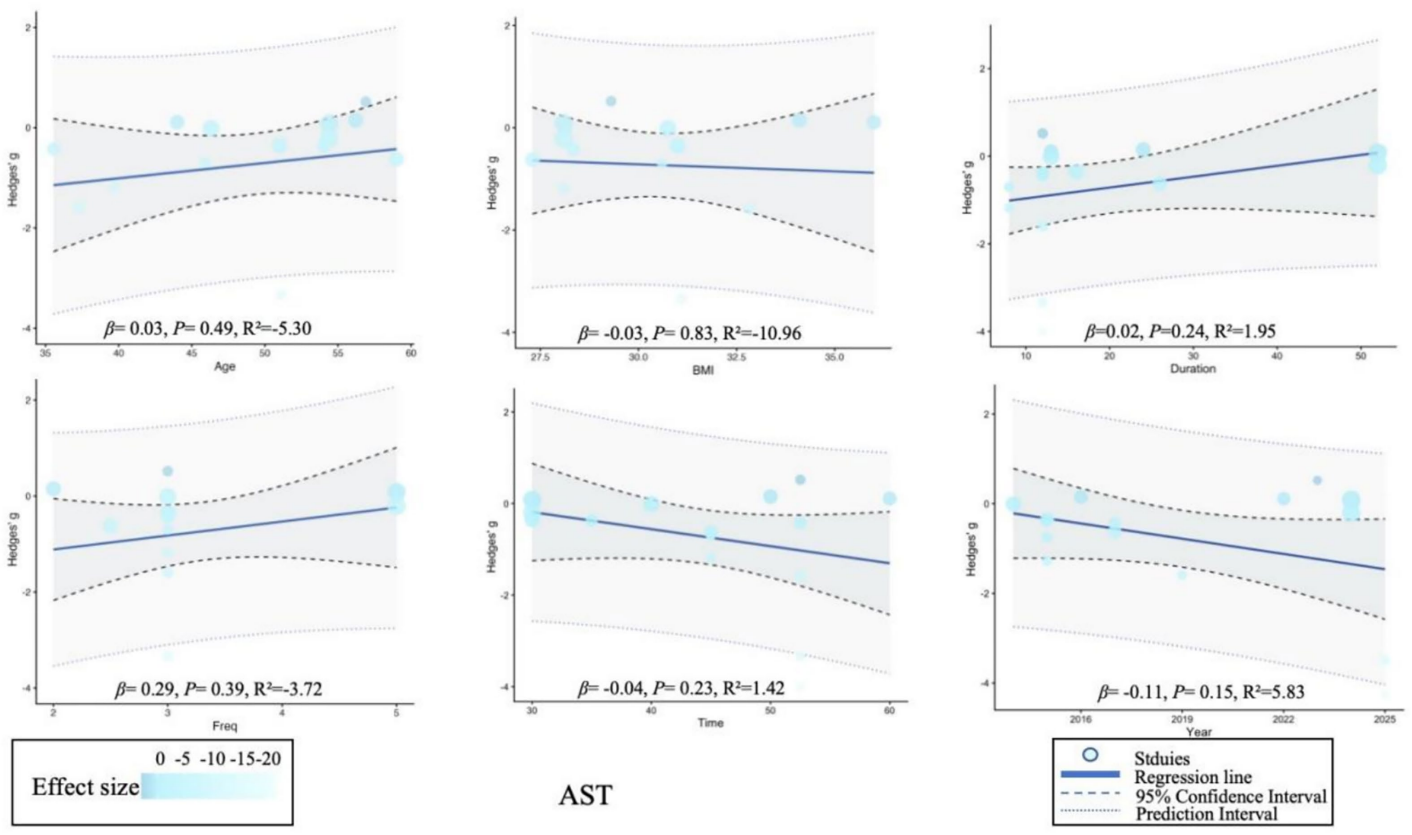
Meta-regression of the effect of exercise on aspartate aminotransferase. *β* (slope), regression coefficient (slope); *p*, *p*-value for statistical significance; *R*^2^, coefficient of determination (model fit).

**Figure 7:**
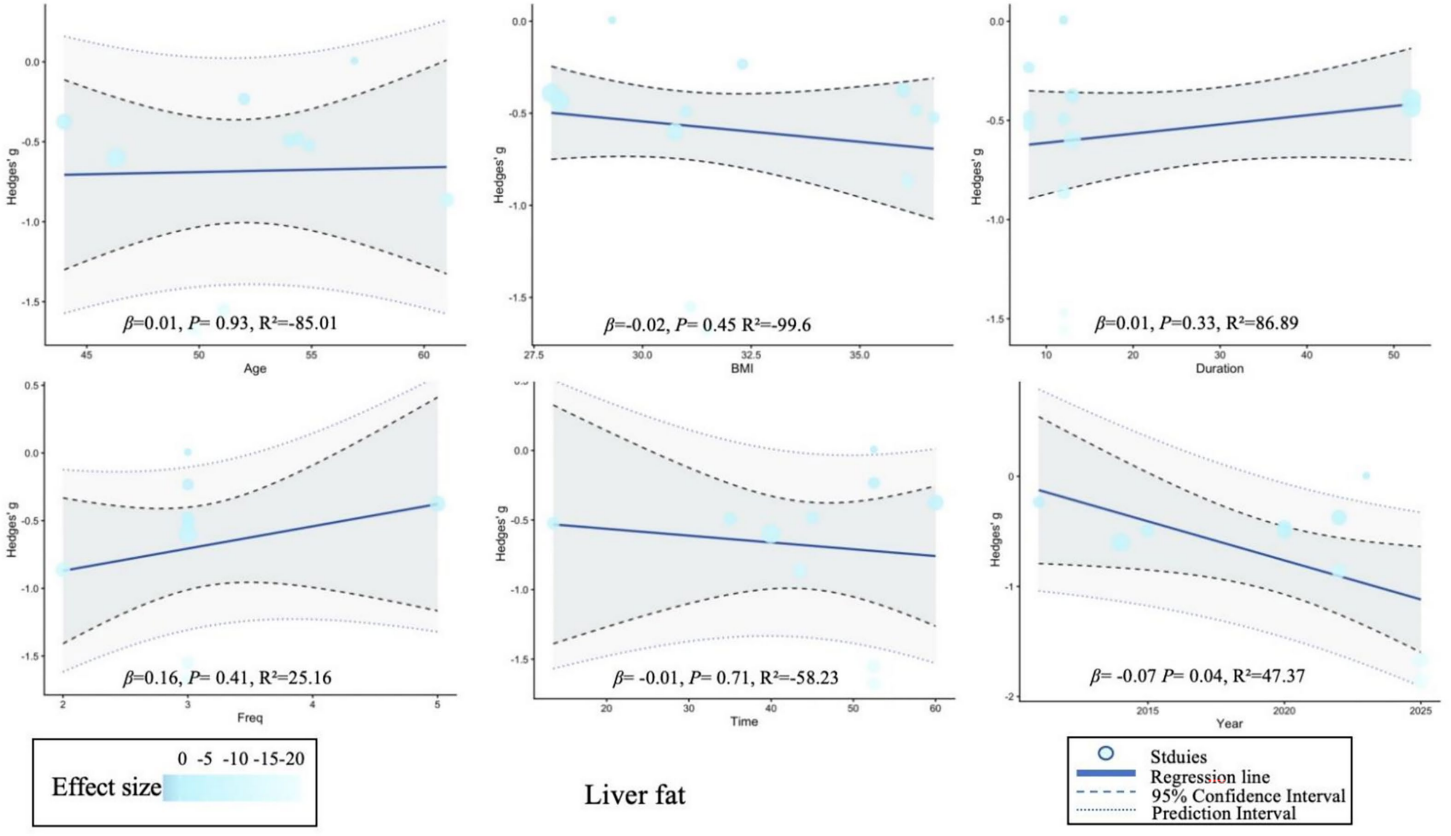
Meta-regression of the effect of exercise on liver fat. *β* (slope), regression coefficient (slope); *p*, *p*-value for statistical significance; *R*^2^, coefficient of determination (model fit).

Meta-regression analysis showed that BMI was significantly correlated with age (*p* = 0.02), while no significant changes were observed in other variables. Due to the limited number of studies on liver stiffness, regression analysis could not be performed on this indicator. For detailed information, please refer to Supplementary Figures A2–6 and Figures 5–7.

### 3.7 Sensitivity analysis

The results of the sensitivity analysis showed that after sequentially excluding each study, the combined effect sizes for all indicators remained stable, the overall effect direction did not change, and the confidence intervals did not cross the null line, indicating that the exclusion of any single study did not significantly affect the overall results (Supplementary Figures A7–A15). Overall, the effect sizes for all indicators were consistent in the sensitivity analysis, further validating the robustness of the findings in this study.

### 3.8 Publication bias analysis

**Figure 8:**
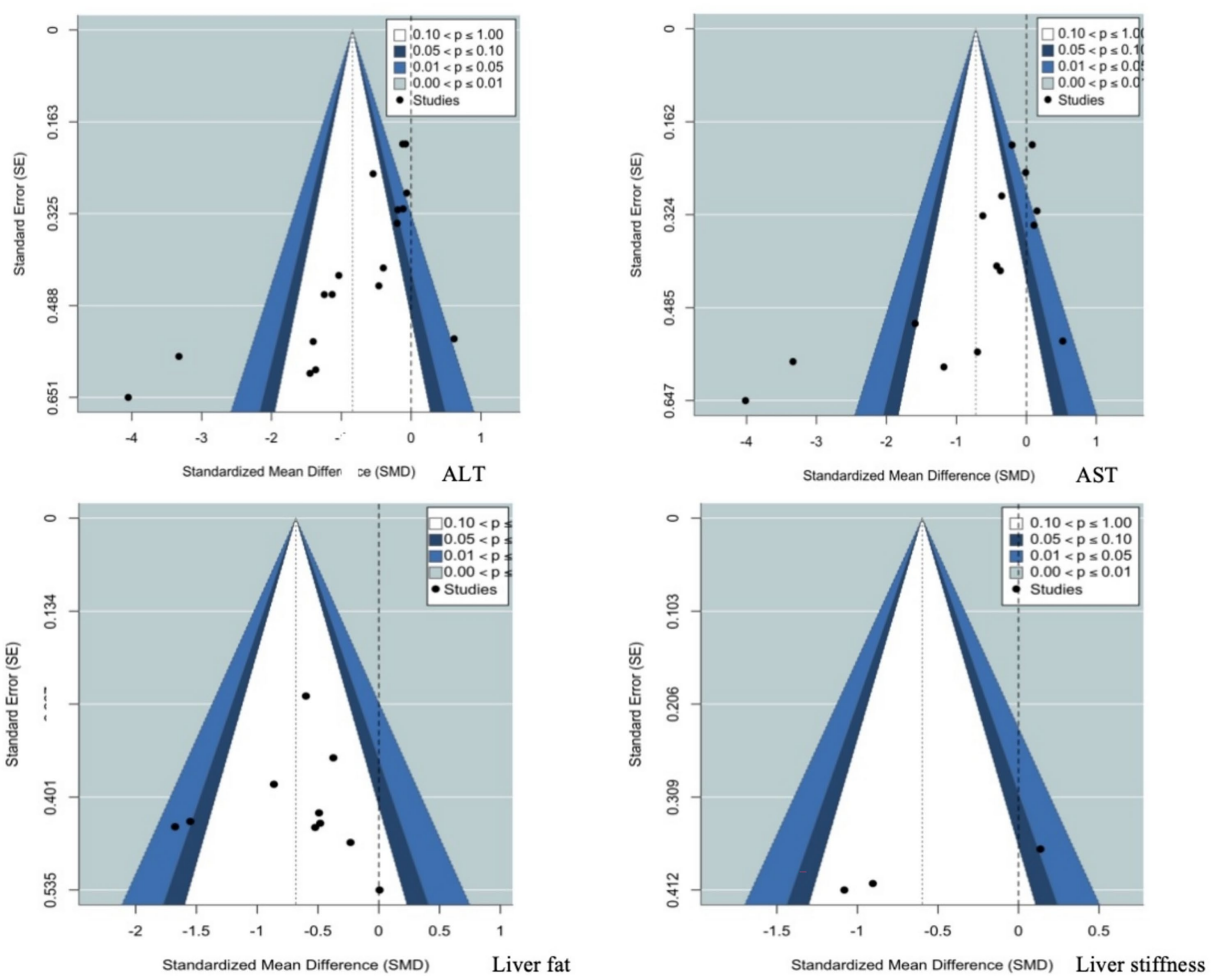
Funnel plot of the effect of exercise on liver function and the progression of fatty liver indicators. ALT, alanine aminotransferase; AST, aspartate aminotransferase.

Funnel plots for the primary outcome measures (Figure 8) showed some asymmetry for ALT, AST, liver fat content, and liver stiffness. Statistical test results indicated significant publication bias for ALT (Egger’s test *p =* 0.01, Begg’s test *p =* 0.01) and AST (Egger’s test *p =* 0.01, Begg’s test *p =* 0.01), while no bias was found for liver fat content (Egger’s test *p =* 0.59, Begg’s test *p =* 0.85). Publication bias tests were not conducted for liver stiffness due to the limited number of studies. After applying the trim-and-fill method to adjust for bias in ALT and AST, substantial changes in the results were observed, suggesting that the credibility of these studies is lower. Therefore, caution should be exercised when interpreting these findings (Supplementary Figures A16–A17).

Funnel plots for secondary outcome measures (Supplementary Figures A19–A23) showed no significant publication bias for BMI (Egger’s test *p* = 0.14, Begg’s test *p* = 0.56), weight (Egger’s test *p =* 0.41, Begg’s test *p =* 0.23), visceral fat (Egger’s test *p =* 0.12, Begg’s test *p =* 0.35), and HbA1c% (Egger’s test *p =* 0.93, Begg’s test *p =* 0.71). However, significant publication bias was found for HOMA-IR (Egger’s test *p =* 0.01, Begg’s test *p =* 0.01). After applying the trim-and-fill method to adjust for bias in HOMA-IR, the results did not change, indicating that the conclusions for this indicator are relatively robust (Supplementary Figure A19).

## 4 Discussion

The results of this study included several RCTs, and the data were based on a large cohort of NAFLD patients confirmed by biopsy and carefully conducted liver histology and imaging examinations. The study found relatively consistent evidence that NAFLD patients who participated in exercise were significantly more likely to reduce their liver enzyme levels and related metabolic parameters. However, for fatty liver progression, due to limited research data, improvements in liver stiffness have not been fully confirmed; however, liver fat content showed a significant reduction. The intervention effects varied across different exercise types, genders, and regions.

Multiple human trials have shown that various exercise regimens can reduce serum ALT and AST levels (7, 8). This is consistent with the evidence from this study. Long-term exercise not only improves liver fat metabolism but also enhances liver function recovery by activating more complex metabolic pathways (25). The mechanisms underlying long-term exercise improvements in liver function include mitochondrial biogenesis activation, increased oxidative capacity in muscle tissue, and modulation of inflammatory pathways that influence hepatic metabolism (26). Increased exercise duration and intensity may trigger adaptive responses to mitochondrial function, insulin sensitivity, and inflammatory markers, which together improve liver function more effectively over time (27). Exercise also reduces inflammation and modulates the expression of pro-inflammatory cytokines, which play a significant role in NAFLD progression. Long-term exercise has been shown to enhance adiponectin levels, decrease tumor necrosis factor-alpha (TNF-α), and reduce hepatic macrophage infiltration, all contributing to improved hepatic function (28). Although short-term exercise interventions show improvement in ALT and AST levels, long-term exercise interventions can further enhance these effects by increasing the body’s adaptation to exercise and the cumulative benefits of metabolic improvements (29). In contrast, a meta-analysis of randomized trials by Keating et al. failed to detect an effect of exercise on ALT, possibly due to differences in the study design (30). First, Keating et al. (30) included healthy individuals in their analysis, whereas this study exclusively focused on NAFLD patients, who generally have more impaired liver function and may experience more pronounced effects from exercise. Second, the duration of the interventions in Keating et al.’s (30) study was generally shorter than ours (with many studies including interventions lasting less than 8 weeks), while this study only included interventions of at least 8 weeks, which may better allow for significant metabolic adaptations and improvements in liver enzyme levels. Another meta-analysis showed that exercise is beneficial for liver fat but does not have a beneficial effect on ALT and AST levels and did not provide data on other enzyme markers (7). Some of the studies included in this analysis may have involved patients with baseline ALT and AST levels that were already within normal ranges, leading to minimal changes in these biomarkers after intervention (31, 32).

In this study, the intervention effects varied across different exercise types, genders, and regions, with HIIT demonstrating more significant results. HIIT can more effectively improve AST and ALT levels in NAFLD patients by activating the sympathetic nervous system, increasing fat oxidation, and enhancing insulin sensitivity (33). The effectiveness of HIIT may stem from its unique physiological and metabolic mechanisms. First, HIIT significantly elevates the heart rate and activates the sympathetic nervous system through high-intensity intermittent exercise, inducing a high metabolic state that facilitates the rapid mobilization of fat stores and enhances fatty acid oxidation, particularly in the liver and muscle tissues (34). This high metabolic state not only accelerates fat burning but also further reduces fat during the post-exercise excess oxygen consumption phase. Additionally, HIIT notably improves mitochondrial function, promotes mitochondrial biogenesis, and increases the fatty acid oxidation capacity of muscle cells, which reduces liver fat accumulation (35, 36). Long-term HIIT training improves insulin sensitivity and enhances the expression of glucose transporters, further improving hepatic metabolic function (37). Compared to traditional AT, HIIT, through short bursts of high-intensity exercise, results in more pronounced fat reduction and greater metabolic improvements, especially in terms of reducing liver fat (35).

Gender differences may also affect exercise outcomes. Men, due to higher muscle mass and lower body fat percentage, are more likely to see improvements in metabolism and liver function (38). In contrast, the impact of estrogen levels on the fat metabolism process and responses in women leads to differences in fat distribution and metabolic characteristics compared to men. Specifically, estrogen affects multiple aspects of fat metabolism, potentially resulting in the accumulation of more fat in the abdominal and lower body regions, which, in turn influences metabolic processes such as liver fat metabolism, thereby altering fat metabolism responses in women (39). Additionally, regional differences may influence the effects of exercise interventions in NAFLD. These differences likely reflect a combination of genetic, environmental, cultural, and lifestyle factors rather than geography (40). For example, ethnic and genetic variations can affect body fat distribution, insulin sensitivity, and lipid metabolism, all of which are known to modulate responses to exercise (41). Distinct dietary patterns across regions—such as higher consumption of saturated fats and refined sugars in some Western populations versus diets with different macronutrient compositions in Asian populations—can influence baseline metabolic risk and the magnitude of change induced by exercise (42). Diets rich in fat and sugar can accelerate the progression of NAFLD, thereby enhancing the effectiveness of exercise, while low-fat and low-sugar diets may slow the progression of the disease (41). Moreover, a sedentary lifestyle can increase insulin resistance, which may amplify the benefits of exercise (43).

The direct mechanisms by which exercise affects liver fat and serum liver enzymes are not yet fully understood. This study demonstrates that exercise significantly improves liver fat in NAFLD patients, but no significant improvement was observed in liver stiffness. Previous studies have consistently shown that exercise effectively improves liver fat. Elisabetta’s research showed that both AT and RT effectively reduced liver fat accumulation, improving liver health and metabolic status (44). Whitsett et al. (10) assessed over 6,000 NAFLD patients, and the results indicated that exercise significantly improved liver fat content, which aligns with the findings of this study. However, the effect of exercise on liver stiffness has been more limited and remains controversial. Most studies report a significant effect of exercise on liver fat, but the impact on liver stiffness is weaker, particularly in short-term interventions. Shojaee-Moradie et al. (29) found that although exercise reduced liver fat, the effect on fibrosis improvement was not significant. This may be because reversing fibrosis requires longer intervention periods, and exercise has a weaker effect on improving fibrosis (18). In NAFLD, liver stiffness is typically normal or mildly elevated, especially in the early stages of the disease. This is often due to the relatively low degree of fibrosis present, as NAFLD usually begins with simple steatosis (fat accumulation in the liver) without significant liver scarring (45). In these cases, liver stiffness may not be significantly altered, which could explain why some studies focusing exclusively on NAFLD patients did not observe significant effects of exercise on liver stiffness (46). Long-term exercise may activate autophagy and increase the expression of matrix metalloproteinases (MMPs), which could aid in remodeling the extracellular matrix and reversing fibrosis (47). Some studies also suggest that improving liver stiffness typically requires a combination of pharmacological interventions and lifestyle changes (10). Several studies suggest that combining exercise with other interventions, such as dietary modification or comprehensive lifestyle changes, may yield greater improvements in hepatic biomarkers and overall liver health than exercise alone. For example, joint diet–exercise strategies have been shown to produce larger reductions in ALT, AST, and HOMA-IR than either intervention alone, indicating that combined interventions may be more effective in ameliorating liver injury in NAFLD patients (48).

Another study found that long-term regular exercise significantly improved the metabolic indicators of NAFLD patients, particularly body weight, visceral fat, BMI, and insulin resistance index, which is consistent with the findings of this research (11). Longer intervention periods enhance the cumulative effects of exercise on muscle mass, insulin sensitivity, and fat oxidation, all of which contribute to significant reductions in metabolic risk factors (49). Amare et al. (50) reported that long-term regular exercise significantly improved both the BMI and lipid profiles in middle-aged men, enhancing metabolic health, especially for markers such as weight and BMI. Meanwhile, Hofataetter observed that long-term exercise can break through the body’s “adaptation plateau,” bringing significant health benefits (51). Furthermore, the subgroup analysis revealed that NAFLD patients without comorbid diabetes experienced a more significant reduction in BMI than those with comorbid diabetes. This could be attributed to the fact that diabetic patients often have higher insulin levels and lower insulin sensitivity, which may restrict fat mobilization and metabolism, diminishing the impact of exercise interventions on BMI. However, the observed correlation between age and BMI improvement may reflect age-related differences in baseline body composition and how exercise influences these changes. With advancing age, muscle mass decreases (sarcopenia) and is often accompanied by an increase in fat, a process that alters overall body composition and metabolic responses to physical activity, making BMI more sensitive to intervention effects in older adults than in younger individuals (52). Age-related reductions in muscle mass (sarcopenia) and the simultaneous increase in fat mass have been widely documented, suggesting that changes in body composition throughout the lifespan may modify the effect of exercise on BMI in older adults. While the specific mechanisms are still under investigation, this “body composition remodeling” makes BMI more responsive to exercise changes in the elderly (53). Although exercise significantly improved metabolic indicators such as visceral fat and insulin resistance, HbA1c% did not show significant improvement. Previous research indicates that significant improvements in HbA1c usually require longer durations or higher-intensity exercise (54). In a 16-week home-based RT trial, patients with type 2 diabetes showed significant improvements in HbA1c, especially under higher-frequency and higher-intensity interventions (55). Several published studies support that structured long-term exercise interventions (e.g., ≥12 weeks) improve glycemic control and reduce HbA1c in adults with type 2 diabetes, with greater benefits observed with higher volumes and higher intensity exercise (54, 56). Specifically, structured exercise ≥150 min/week is associated with larger HbA1c declines, and meta-analytic evidence shows that higher-intensity resistance exercise produces greater reductions in HbA1c than lower-intensity exercise, consistent with a dose–response relationship between training intensity/duration and glycemic improvement (57).

Long-term regular exercise significantly reduced ALT, AST, and liver fat levels, while improving body weight, BMI, insulin resistance index, and visceral fat. However, no significant changes were observed in liver stiffness or HbA1c. The effectiveness of the intervention was influenced by the type of exercise, particularly in relation to the body composition of NAFLD patients. Given the variations in intervention protocols, potential publication bias, and the small sample sizes in studies on liver stiffness, the conclusions should be interpreted with caution.

### 4.1 Limitations

Although this study strictly followed the systematic review guidelines, and the results are highly reliable, there are still some limitations. First, the quality of the studies varied considerably. Despite using a random-effects model and conducting sensitivity analyses, substantial heterogeneity across studies suggests that differences in participant characteristics, intervention methods, and outcome measures may have contributed to the observed results. Second, the sample sizes for studies related to liver stiffness were relatively small, resulting in insufficient statistical power and limiting the ability to fully capture the effects of exercise on liver fibrosis improvement. Third, some studies did not provide detailed descriptions of the supervision methods (e.g., professional guidance and adherence monitoring), which may have led to variations in the implementation of interventions and introduced a potential ROB1. Finally, although corrections were made using the adjustment method, Egger’s test for ALT and AST did not pass, indicating potential bias that may affect the reliability of these two indicators. Future research should conduct large-scale, multi-center RCTs with standardized exercise protocols and longer follow-up periods to confirm the long-term effects of exercise on NAFLD. Exploring the combined effects of exercise with other lifestyle changes, such as diet, will also help identify the most effective multi-component intervention strategies.

## 5 Conclusion

This meta-analysis demonstrates that long-term, regular exercise significantly improves ALT, AST, and liver fat content in NAFLD patients, along with body weight, BMI, VAT, and HOMA-IR. However, improvements in liver stiffness and HbA1c% are relatively limited. Subgroup analyses identified exercise type, gender, and region as moderators of the intervention effects, while regression analysis showed a significant correlation between BMI and age, with no significant correlations for other variables. Nevertheless, the findings may be interpreted with caution, as the statistical power of this meta-analysis is limited by small sample sizes and the limited number of studies available for certain outcomes, particularly liver stiffness, for which only three studies contributed data.

## Data Availability

The original contributions presented in the study are included in the article/Supplementary material, further inquiries can be directed to the corresponding author.
