# Supplementary material for: The impact of long-term exercise on liver function, fatty liver progression, and related metabolic markers in NAFLD patients: a meta-analysis of randomized controlled trials
Source: Front Nutr. 2026 Mar 30;13:1731510. doi: 10.3389/fnut.2026.1731510 (PMC13070912; doi:10.3389/fnut.2026.1731510)
Supplement: Supplementary file 1 [file Supplementary_file_1.docx]

Supplementary Material

# Supplementary Materials 1 Search strategy

Pubmed

Table A1 PubMed Search Terms

| Search | Query |
| --- | --- |
| #1 | "Exercise"[Mesh] Sort by: Most Recent |
| #2 | (((((((((((((((((((((Exercise, Physical[Title/Abstract]) OR (Exercises, Physical[Title/Abstract])) OR (Exercise[Title/Abstract])) OR (Exercises[Title/Abstract])) OR (Exercise, Aerobic[Title/Abstract])) OR (Aerobic Exercise[Title/Abstract])) OR (Aerobic Exercises[Title/Abstract])) OR (Exercises, Aerobic[Title/Abstract])) OR (Exercise, Isometric[Title/Abstract])) OR (Exercises, Isometric[Title/Abstract])) OR (Isometric Exercises[Title/Abstract])) OR (Isometric Exercise[Title/Abstract])) OR (Isometric Exercise[Title/Abstract])) OR (Acute Exercise[Title/Abstract])) OR (Acute Exercise[Title/Abstract])) OR (Exercise, Acute[Title/Abstract])) OR (Exercises, Acute[Title/Abstract])) OR (Exercise Training[Title/Abstract])) OR (Exercise Trainings[Title/Abstract])) OR (Physical Activity[Title/Abstract])) OR (Activities, Physical[Title/Abstract])) OR (Physical Activities[Title/Abstract]) |
| #3 | #1 OR #2 |
| #4 | "Non-alcoholic Fatty Liver Disease"[Mesh] Sort by: Most Recent |
| #5 | ((((((((((((((Nonalcoholic Fatty Liver Disease[Title/Abstract]) OR (Fatty Liver, Nonalcoholic[Title/Abstract])) OR (Fatty Livers, Nonalcoholic[Title/Abstract])) OR (Liver, Nonalcoholic Fatty[Title/Abstract])) OR (Livers, Nonalcoholic Fatty[Title/Abstract])) OR (Nonalcoholic Fatty Liver[Title/Abstract])) OR (Nonalcoholic Fatty Livers[Title/Abstract])) OR (NAFLD[Title/Abstract])) OR (MASLD[Title/Abstract])) OR (Nonalcoholic Fatty Liver Disease[Title/Abstract])) OR (Nonalcoholic Steatohepatitis[Title/Abstract])) OR (Nonalcoholic[Title/Abstract])) OR (Steatohepatitis, Nonalcoholic[Title/Abstract])) OR (NASH[Title/Abstract])) |
| #6 | (((("Non-alcoholic Fatty Liver Disease"[Mesh]) OR ((NAFLD OR MAFLD OR MASLD OR NASH OR "nonalcoholic fatty liver disease" OR "metabolic dysfunction-associated fatty liver"))) AND ("Exercise"[Mesh])) OR ((((((((((((((((((((((high intensity interval training[Title/Abstract]) OR (moderate intensity continuous training[Title/Abstract])) OR (HIIT[Title/Abstract])) OR (MICT[Title/Abstract])) OR (AE[Title/Abstract])) OR (AT[Title/Abstract])) OR (RE[Title/Abstract])) OR (RT[Title/Abstract])) OR (combine[Title/Abstract])) OR (aerobic exercise[Title/Abstract])) OR (aerobic training[Title/Abstract])) OR (resistance exercise[Title/Abstract])) OR (resistance training[Title/Abstract])) OR (combined training[Title/Abstract])) OR (concurrent training[Title/Abstract])) OR (mixed exercise[Title/Abstract])) OR (mixed training[Title/Abstract])) OR (exercise[Title/Abstract])) OR (exercise therapy[Title/Abstract])) OR (exercise intervention[Title/Abstract])) OR (exercise program[Title/Abstract])) OR (physical activity[Title/Abstract]))) AND ((RCT[Title/Abstract]) OR (randomized controlled trial[Title/Abstract])) |
| #7 | (RCT[Title/Abstract]) OR (randomized controlled trial [Title/Abstract]) |
| #8 | #4 OR #5 |
| #9 | 7 AND #8 |
| #10 | #9 AND #3 |

Embase

Table A2 Embase Search Terms

| Search | Query |
| --- | --- |
| #1 | 'exercise'/exp OR 'exercise' |
| #2 | 'exercises':ab,ti OR 'exercise, physical':ab,ti OR 'exercises, physical':ab,ti OR 'physical exercise':ab,ti OR 'physical exercises':ab,ti OR 'exercise, aerobic':ab,ti OR 'aerobic exercise':ab,ti OR 'aerobic exercises':ab,ti OR 'exercises, aerobic':ab,ti OR 'exercise, isometric':ab,ti OR 'exercises, isometric':ab,ti OR 'isometric exercises':ab,ti OR 'isometric exercise':ab,ti OR 'acute exercise':ab,ti OR 'acute exercises':ab,ti OR 'exercise, acute':ab,ti OR 'exercises, acute':ab,ti OR 'exercise training':ab,ti OR 'exercise trainings':ab,ti OR 'training, exercise':ab,ti OR 'trainings, exercise':ab,ti OR 'physical activity':ab,ti OR 'activities, physical':ab,ti OR 'activity, physical':ab,ti OR 'physical activities':ab,ti OR 'biometric exercise':ab,ti |
| #3 | #2 OR #3 |
| #4 | 'nonalcoholic fatty liver'/exp OR 'nonalcoholic fatty liver' |
| #5 | 'nafld (nonalcoholic fatty liver disease)':ab,ti OR 'non alcoholic fatty liver disease;':ab,ti OR 'non alcoholic hepato-steatosis':ab,ti OR 'non alcoholic hepatosteatosis':ab,ti OR 'non alcoholic liver steatosis':ab,ti OR 'non alcoholic steatotic hepatopathy':ab,ti OR 'non-alcoholic fatty liver':ab,ti OR 'non-alcoholic fatty liver disease':ab,ti OR 'non-alcoholic hepatic steatosis':ab,ti OR 'non-alcoholic fld':ab,ti OR 'nonalcoholic fatty liver disease':ab,ti OR 'nonalcoholic fld':ab,ti OR 'nonalcoholic hepatic steatosis':ab,ti OR 'nonalcoholic liver steatosis':ab,ti OR 'nonalcoholic hepatosteatosis':ab,ti OR 'nonalcoholic fatty liver':ab,ti |
| #6 | #5 OR #6 |
| #7 | #4 AND #7 |

Web of science

Table A3 Web of Science Search Terms

| Search | Query |
| --- | --- |
| #1 | Exercise OR Training OR “Exercise Physical”OR “Physical Exercise” OR Aerobic OR “Aerobic Exercises” OR Exercise, Isometric OR “Isometric Exercises” OR “Acute Exercise” OR Exercise Training OR “Physical Activity”OR “Physical Activities”OR “biometric exercise”OR “exercise capacity” OR “exercise performance” OR “fitness training”OR “fitness workout” OR “physical conditioning” OR “physical effort” OR “physical exercise” OR “physical work-out” OR exercise |
| #2 | “fibrosis index” OR “NAFLD”OR “fibrosis score” OR “Fibrosis Scoring”OR “non alcoholic fatty liver disease” OR “Non Alcoholic Fatty Liver Disease” OR “Fibrosis Score” OR “Non-Alcoholic liver fatty liver disease fibrotic score OR ” OR “Fatty Liver” OR Nonalcoholic OR “Fatty Livers”OR Nonalcoholic Fatty Liver OR “Nonalcoholic Fatty Livers” OR “NAFLD”OR Nonalcoholic OR “MASLD” OR “NASH” |
| #3 | #1 AND #2 |

Cochrane Library

Table A4 Cochrane Library Search Terms

| Search | Query |
| --- | --- |
| #1 | MeSH descriptor: [Exercise] explode all trees |
| #2 | (Activities,):ti,ab,kw or (Physical Activities;):ti,ab,kw or (Exercises, Isometric;):ti,ab,kw or ( Isometric Exercise;):ti,ab,kw or (Exercise Training;):ti,ab,kw or (Trainings, Exercise;):ti,ab,kw or (Aerobic Exercise;):ti,ab,kw or (Exercises, Aerobic;):ti,ab,kw or (Acute Exercises;):ti,ab,kw or (Exercises, Acute;):ti,ab,kw or (Physical Exercises;):ti,ab,kw or (Exercises; ):ti,ab,kw |
| #3 | #1 OR #2 |
| #4 | (Fatty Liver,):ti,ab,kw or (Nonalcoholic;):ti,ab,kw or (Nonalcoholic Fatty Liver;):ti,ab,kw or (Non alcoholic Fatty Liver Disease;):ti,ab,kw or (Nonalcoholic Fatty;):ti,ab,kw or (Nonalcoholic Fatty Liver Disease;):ti,ab,kw or (Liver, Nonalcoholic Fatty;):ti,ab,kw or (NAFLD;):ti,ab,kw or (Nonalcoholic Fatty Livers;):ti,ab,kw or (Nonalcoholic Steatohepatitides;):ti,ab,kw or (Nonalcoholic Steatohepatitis):ti,ab,kw |
| #5 | #4 OR #5 |
| #6 | #3 AND #6 |

EBSCO

Table A5 EBSCO Search Terms

| Search | Query |
| --- | --- |
| #1 | AB Exercise OR AB Exercise, Physical OR AB Exercises OR AB Exercise Physical OR AB Physical Exercise OR AB Physical Exercises OR AB Aerobic Exercise OR AB Aerobic Exercises OR AB Isometric Exercises OR AB Isometric Exercise OR AB Acute Exercise OR AB Acute Exercises OR AB Exercises Acute OR AB Exercise Training OR AB Exercise Trainings OR AB Physical Activities OR AB Physical Activity OR AB biometric exercise OR AB exercise capacity OR AB exercise performance OR AB exercise training OR AB fitness training OR AB fitness workout OR AB physical conditioning OR AB physical work-out OR AB physical workout |
| #2 | AB Exercise OR AB Exercise, Physical OR AB Exercises OR AB Exercise Physical OR AB Physical Exercise OR AB Physical Exercises OR AB Aerobic Exercise OR AB Aerobic Exercises OR AB Isometric Exercises OR AB Isometric Exercise OR AB Acute Exercise OR AB Acute Exercises OR AB Exercises Acute OR AB Exercise Training OR AB Exercise Trainings OR AB Physical Activities OR AB Physical Activity OR AB biometric exercise OR AB exercise capacity OR AB exercise performance OR AB exercise training OR AB fitness training OR AB fitness workout OR AB physical conditioning OR AB physical work-out OR AB physical workout |

Table A6 Overall Risk of Bias Assessment for RCT Studies

| Study | High | No clear | All |
| --- | --- | --- | --- |
| Abdelbasset 2020 | 0 | 2 | High Quality |
| Hoseini 2020 | 0 | 3 | High Quality |
| Guo 2025 | 1 | 2 | Medium Quality |
| Naimimohasses 2022 | 3 | 2 | Low Quality |
| Cuthbertson 2016 | 1 | 3 | Medium Quality |
| Shamsoddini 2015 | 0 | 3 | High Quality |
| Cheng 2016 | 1 | 3 | Medium Quality |
| Ezpeleta 2023 | 0 | 2 | High Quality |
| Mohammadi 2019 | 0 | 5 | Low Quality |
| Zelber-Sagi 2014 | 1 | 2 | Medium Quality |
| Hui-Jie Zhang 2016 | 1 | 1 | Medium Quality |
| Moradi Kelardeh 2017 | 0 | 4 | Medium Quality |
| Sullivan 2012 | 2 | 3 | Low Quality |
| Hallsworth 2011 | 1 | 2 | Medium Quality |
| Csader 2023 | 1 | 4 | Low Quality |
| Rezende 2016 | 0 | 3 | High Quality |
| Hallsworth 2015 | 1 | 3 | Medium Quality |


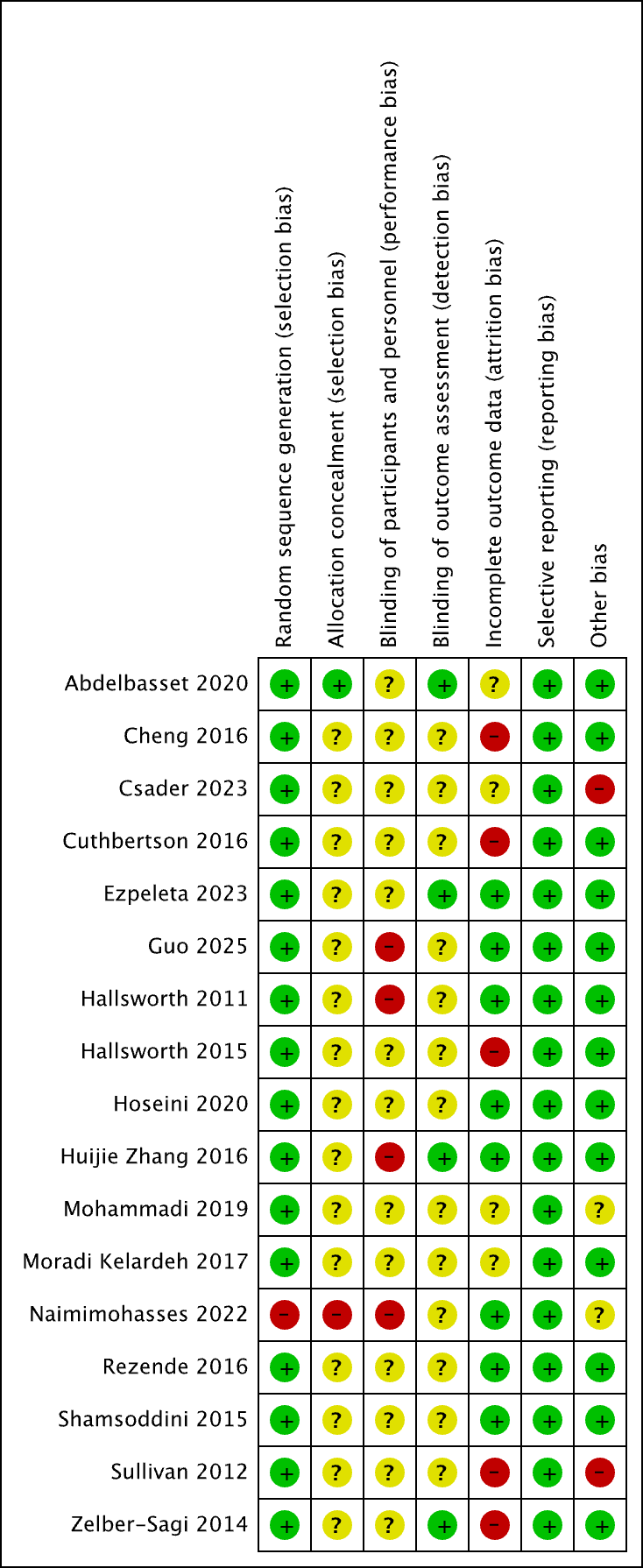


Figure A1 Risk of Bias Assessment for Each Study

Table A7 Subgroup Analysis Table for metabolic index Indicators

| type | subgroups | K | MD/SMD（95%CI） | I | P |
| --- | --- | --- | --- | --- | --- |
| BMI |  |  |  |  |  |
| Type | AT | 8 | -1.1551 [-1.7611; -0.5491] | 31.9 |  |
|  | RT | 4 | -0.2560 [-0.4748; -0.0372] | 0 |  |
|  | HIIT | 4 | -1.4642 [-2.0292; -0.8992] | 0 |  |
|  |  |  |  |  | **<0.001** |
| Continent | Africa | 2 | -2.6034 [-5.5371; 0.3302] | 0 |  |
|  | North America | 1 | -0.1900 [-1.1991; 0.8191] | 0 |  |
|  | South America | 1 | -0.3000 [-3.2317; 2.6317] | - |  |
|  | Asia | 8 | -1.1143 [-1.9328; -0.2958] | 79.8 |  |
|  | Europe | 4 | -0.9015 [-1.5444; -0.2586] | 0 |  |
|  |  |  |  |  | 0.466 |
| Sex | Mix | 8 | -1.0058 [-1.6586; -0.3529] | 78.8 |  |
|  | Male | 5 | -0.4401 [-1.7674; 0.8871] | 0 |  |
|  | Female | 2 | -1.8093 [-3.7781; 0.1596] | 33.0 |  |
|  | No clear | 1 | -0.5000 [-5.5855; 4.5855] | - |  |
|  |  |  |  |  | 0.716 |
| Diabetes Mellitus | Yes | 6 | -0.4700 [-1.3205; 0.3806] |  |  |
|  | No | 4 | -0.3953 [-0.7544; -0.0362] | 14.7 |  |
|  | No clear | 6 | -1.5862 [-1.9969; -1.1754] |  |  |
|  |  |  |  |  | **<0.001** |
| Weight |  |  |  |  |  |
| Type | AT | 10 | -2.1651 [-3.0206; -1.3096] | 51.6 |  |
|  | RT | 2 | -0.7215 [-1.3684; -0.0745] | 0 |  |
|  | HIIT | 2 | -3.9680 [-5.5220; -2.4140] | 0 |  |
|  |  |  |  |  | **<0.001** |
| Continent | North America | 3 | -1.2676 [-1.9246; -0.6106] | 0 |  |
|  | Asia | 8 | -2.2613 [-3.5222; -1.0004] | 78.6 |  |
|  | Europe | 3 | -2.6858 [-4.0227; -1.3489] | 0 |  |
|  |  |  |  |  | 0.105 |
| Sex | Mix | 10 | -2.2795 [ -3.1650; -1.3940] | 74.2 |  |
|  | Male | 2 | -1.2254 [-10.2048; 7.7540] | 0 |  |
|  | Female | 1 | 0.5000 [ -2.8252; 3.8252] | - |  |
|  | No clear | 1 | -1.4000 [-15.4076; 12.6076] | - |  |
|  |  |  |  |  | 0.466 |
| Diabetes Mellitus | Yes | 3 | -1.2701 [-1.9275; -0.6126] | 0 |  |
|  | No | 7 | -1.8192 [-2.7785; -0.8600] | 58.1 |  |
|  | No clear | 4 | -3.1363 [-5.1532; -1.1194] | 57.3 |  |
|  |  |  |  |  | 0.185 |
| VAT |  |  |  |  |  |
| Type | AT | 6 | -0.4915 [-1.0036; 0.0206] | 73.6 |  |
|  | HIIT | 4 | -0.6369 [-1.4244; 0.1507] | 67.0 |  |
|  |  |  |  |  | 0.762 |
| Continent | Africa | 2 | -0.9168 [-1.5556; -0.2780] | 0 |  |
|  | North America | 1 | -0.3676 [-1.0432; 0.3080] | 0 |  |
|  | Asia | 4 | -0.7265 [-1.4667; 0.0137] | 85.4 |  |
|  | Europe | 2 | 0.0637 [-0.5821; 0.7095] | 0 |  |
|  |  |  |  |  | 0.168 |
| Sex | Mix | 7 | -0.6991 [-1.1863; -0.2120] | 74.8 |  |
|  | Female | 1 | -0.0987 [-1.1472; 0.9498] | - |  |
|  | No clear | 1 | 0.1629 [-0.6568; 0.9827] | - |  |
|  |  |  |  |  | 0.168 |
| Diabetes Mellitus | Yes | 4 | -0.5660 [-0.9904; -0.1415] | 0 |  |
|  | No | 2 | -0.1203 [-0.6737; 0.4331] | 74.2 |  |
|  | No clear | 3 | -0.9493 [-2.0716; 0.1730] | 80.9 |  |
|  |  |  |  |  | 0.300 |
| HOMA-IR |  |  |  |  |  |
| Type | AT | 6 | -0.9882 [-1.7708; -0.2055 | 89.1 |  |
|  | RT | 2 | 0.0880 [-0.5453; 0.7213] | 39.6 |  |
|  | HIIT | 3 | -1.3456 [-3.0134; 0.3221] | 93.6 |  |
|  |  |  |  |  | 0.058 |
| Continent | Africa | 2 | -0.6439 [-1.2639; -0.0240] | 0 |  |
|  | North America | 1 | -0.6114 [-1.2978; 0.0750] | - |  |
|  | South America | 1 | 0.0396 [-0.5810; 0.6602] | - |  |
|  | Asia | 4 | -1.9467 [-3.9898; 0.0964] | 96.6 |  |
|  | Europe | 3 | -0.4060 [-0.8253; 0.0132] | 0 |  |
|  |  |  |  |  | 0.282 |
| Sex | Mix | 7 | -1.2184 [-2.1307; -0.3061] | 93.3 |  |
|  | Male | 1 | 0.0396 [-0.5810; 0.6602] | - |  |
|  | Female | 1 | -1.0442 [-1.9913; -0.0970] | - |  |
|  | No clear | 2 | -0.3096 [-0.9227; 0.3035] | 0 |  |
|  |  |  |  |  | 0.078 |
| Diabetes Mellitus | Yes | 4 | -0.3921 [-0.7617; -0.0225] | 0 |  |
|  | No | 2 | -0.0733 [-0.8637; 0.7171] | 77.1 |  |
|  | No clear | 5 | -1.7319 [-3.1140; -0.3497] | 89.3 |  |
|  |  |  |  |  | 0.121 |
| HbA1c% |  |  |  |  |  |
| Type | AT | 4 | -0.0307 [-0.3692; 0.3079] | 0 |  |
|  | RT | 1 | -0.3661 [-0.8606; 0.1285] | - |  |
|  | HIIT | 3 | -0.1987 [-0.7146; 0.3173] | 0 |  |
|  |  |  |  |  | 0.537 |
| Continent | Africa | 2 | -0.4173 [-1.0277; 0.1931] | 0 |  |
|  | North America | 2 | -0.0349 [-0.4910; 0.4213] | 0 |  |
|  | South America | 2 | 0.1615 [-0.4602; 0.7831] | 0 |  |
|  | Asia | 1 | -0.3661 [-0.8606; 0.1285] | - |  |
|  | Europe | 1 | -0.0599 [-0.7049; 0.5851] | - |  |
|  |  |  |  |  | 0.560 |
| Sex | Mix | 5 | -0.2404 [-0.5343; 0.0534] | 0 |  |
|  | Male | 1 | 0.1615 [-0.4602; 0.7831] | - |  |
|  | Female | 1 | -0.0189 [-1.0666; 1.0287] | - |  |
|  | No clear | 1 | -0.0849 [-0.9035; 0.7336] | - |  |
|  |  |  |  |  | 0.703 |
| Diabetes Mellitus | Yes | 6 | -0.0807 [-0.3824; 0.2209] | 0 |  |
|  | No | 1 | -0.3661 [-0.8606; 0.1285] | - |  |
|  | No clear | 1 | -0.0849 [-0.9035; 0.7336] | - |  |
|  |  |  |  |  | 0.619 |

Note: VAT: Visceral Adipose Tissue；HOMA-IR: Homeostasis Model Assessment of Insulin Resistance；HbA1c%: Glycated Hemoglobin Percentage.


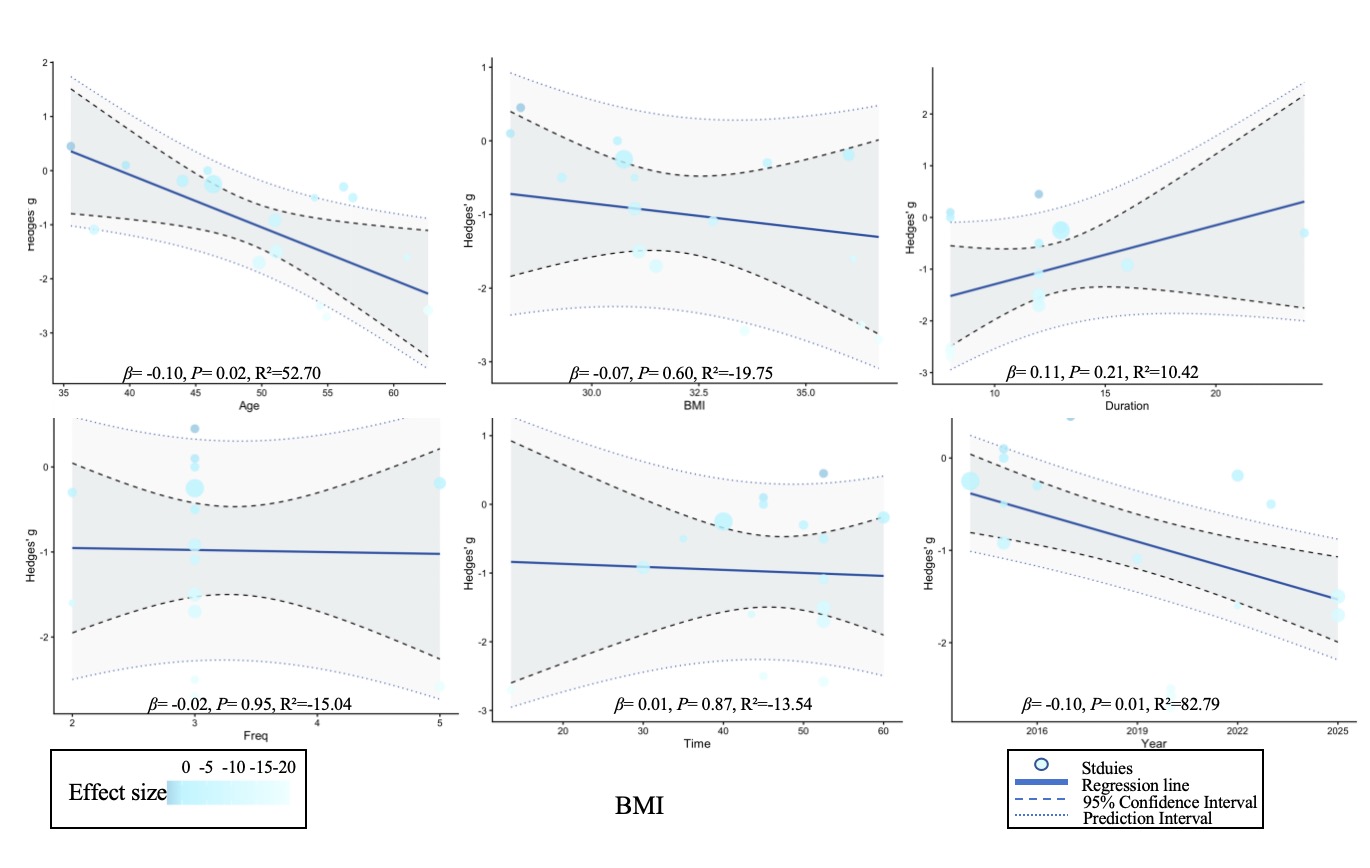


Figure A2 Meta-Regression of the Effect of Exercise on BMI


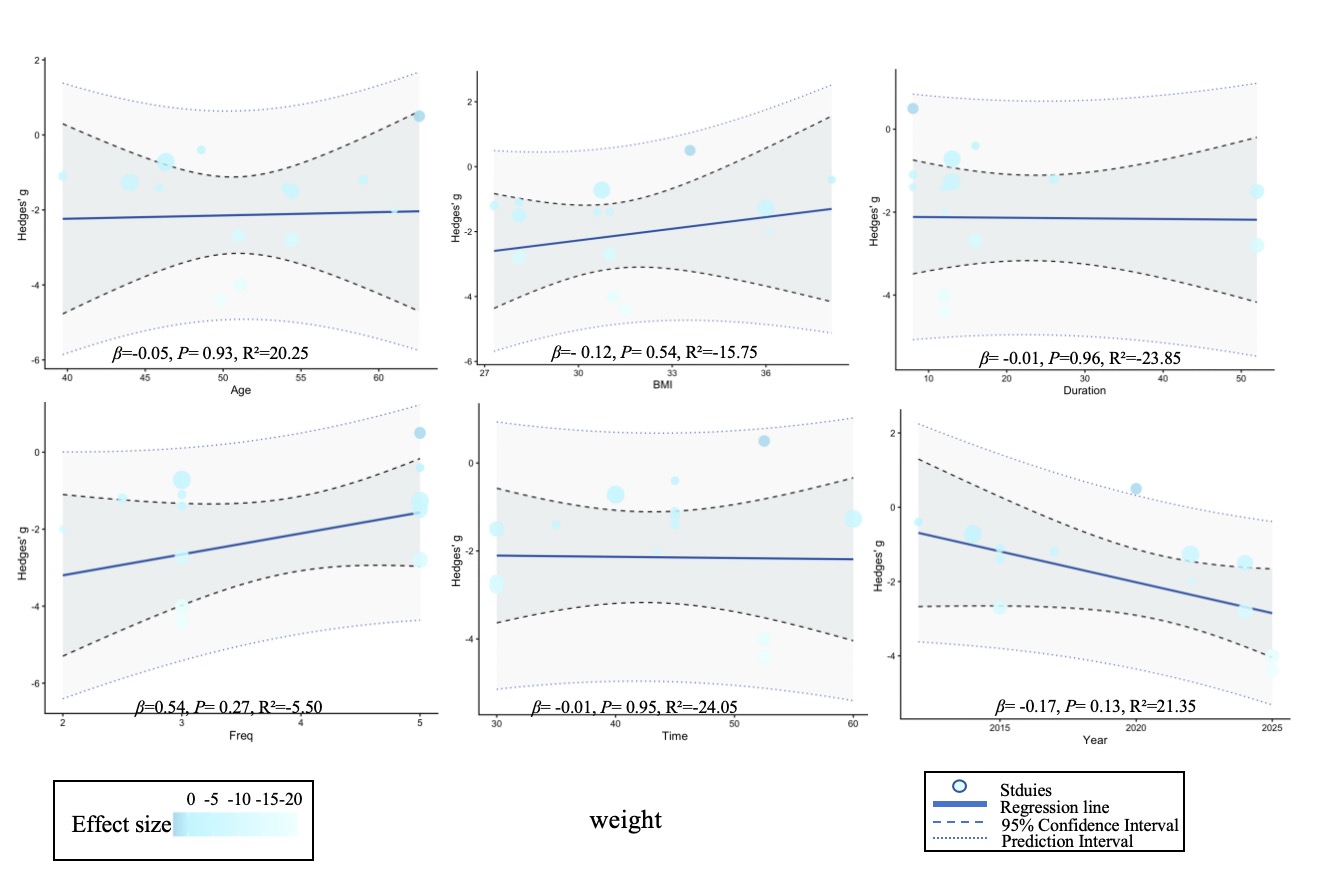


Figure A3 Meta-Regression of the Effect of Exercise on weight


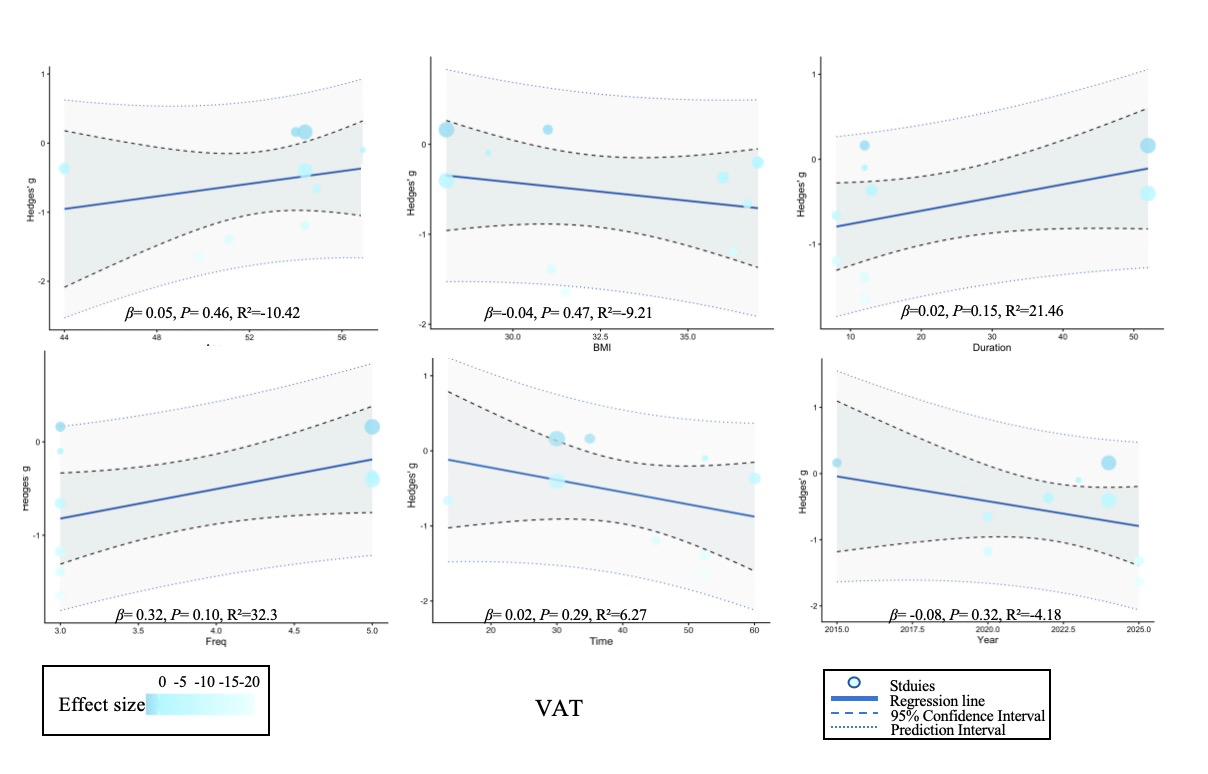


Figure A4 Meta-Regression of the Effect of Exercise on Visceral Adipose Tissue


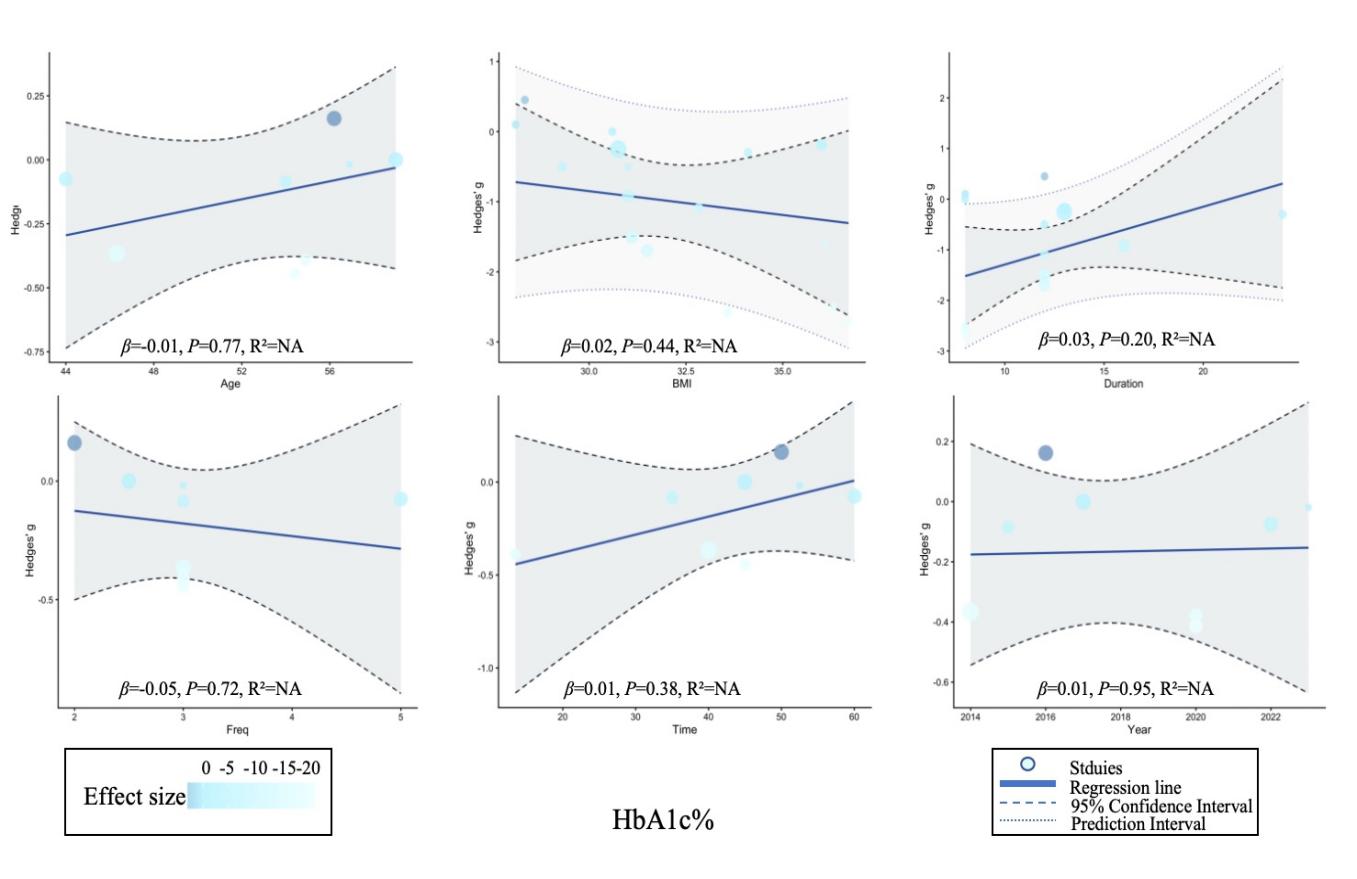


Figure A5 Meta-Regression of the Effect of Exercise on Glycated Hemoglobin Percentage


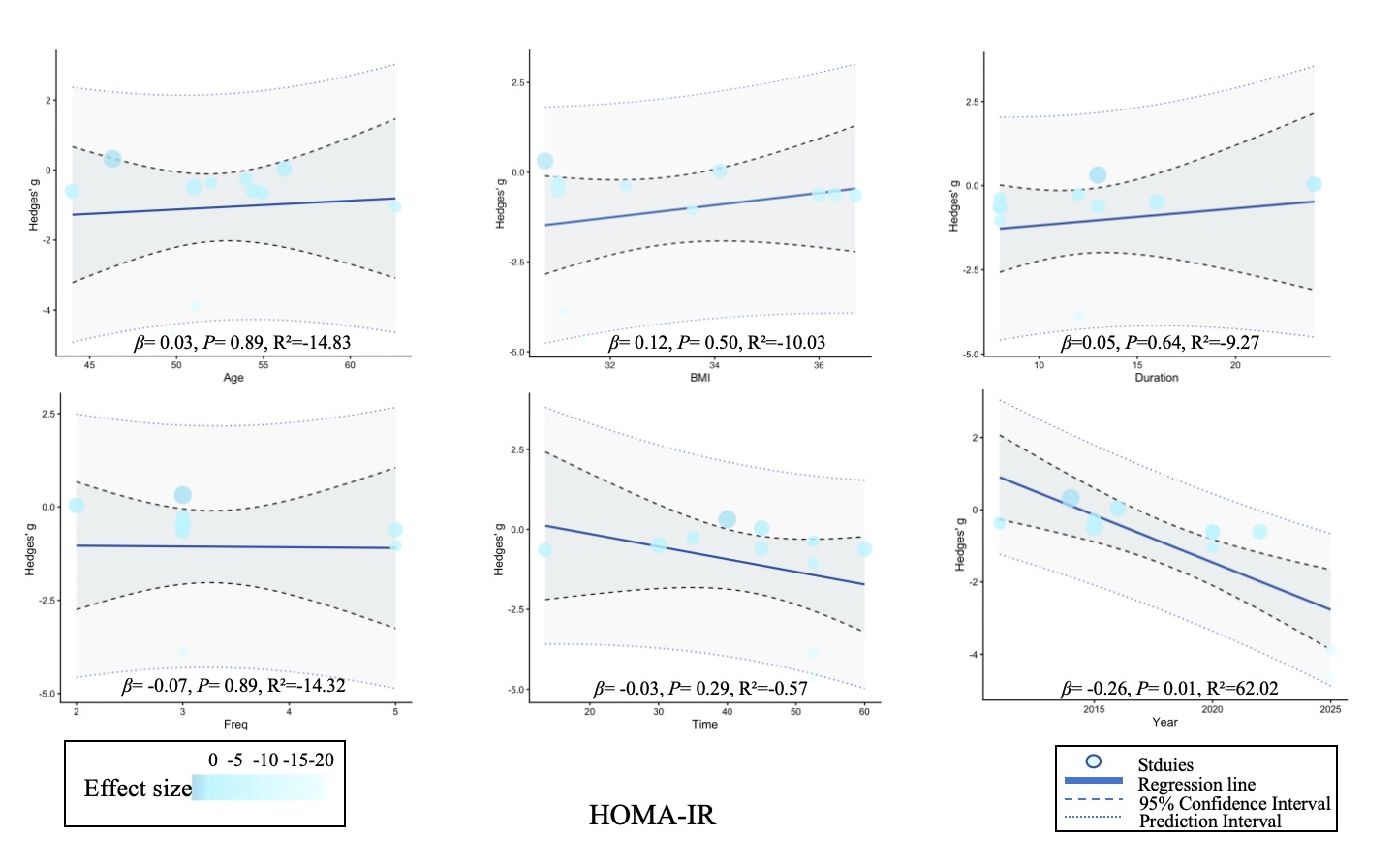


Figure A6 Meta-Regression of the Effect of Exercise on Homeostasis Model Assessment of Insulin Resistance


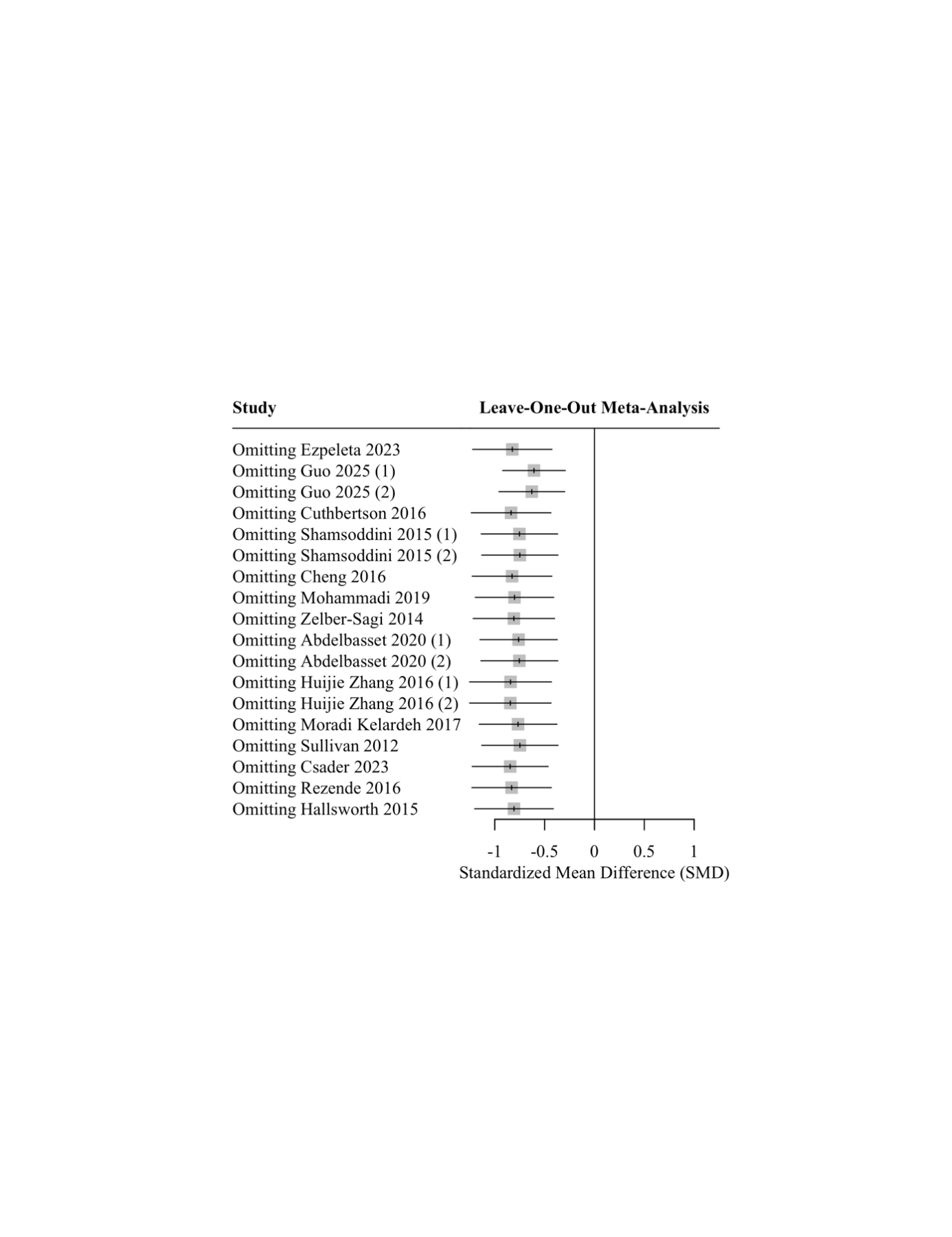


Figure A7 Leave-One-Out Meta-Analysis of Alanine Aminotransferase Levels


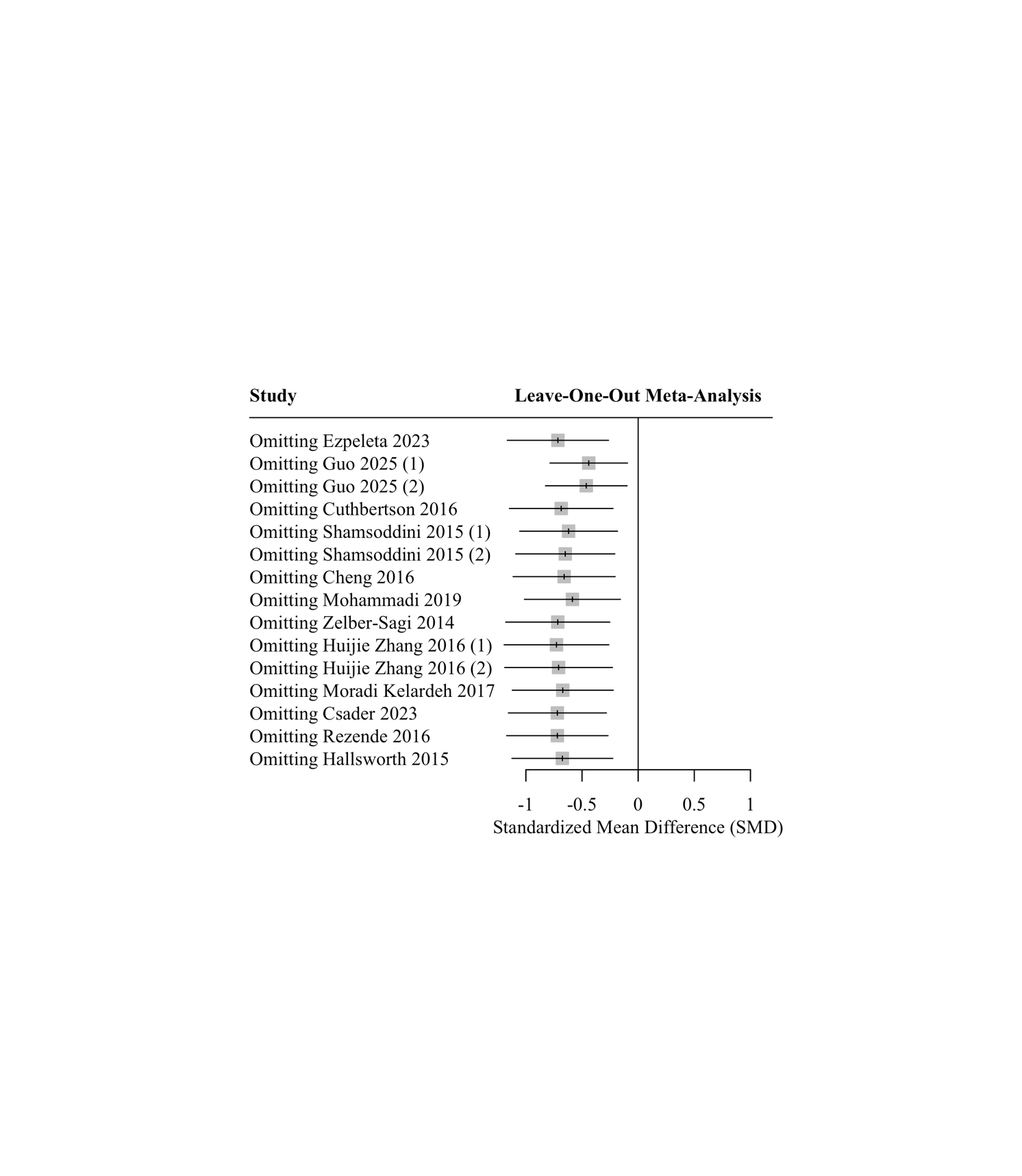


Figure A8 Leave-One-Out Meta-Analysis of Aspartate Aminotransferase Levels


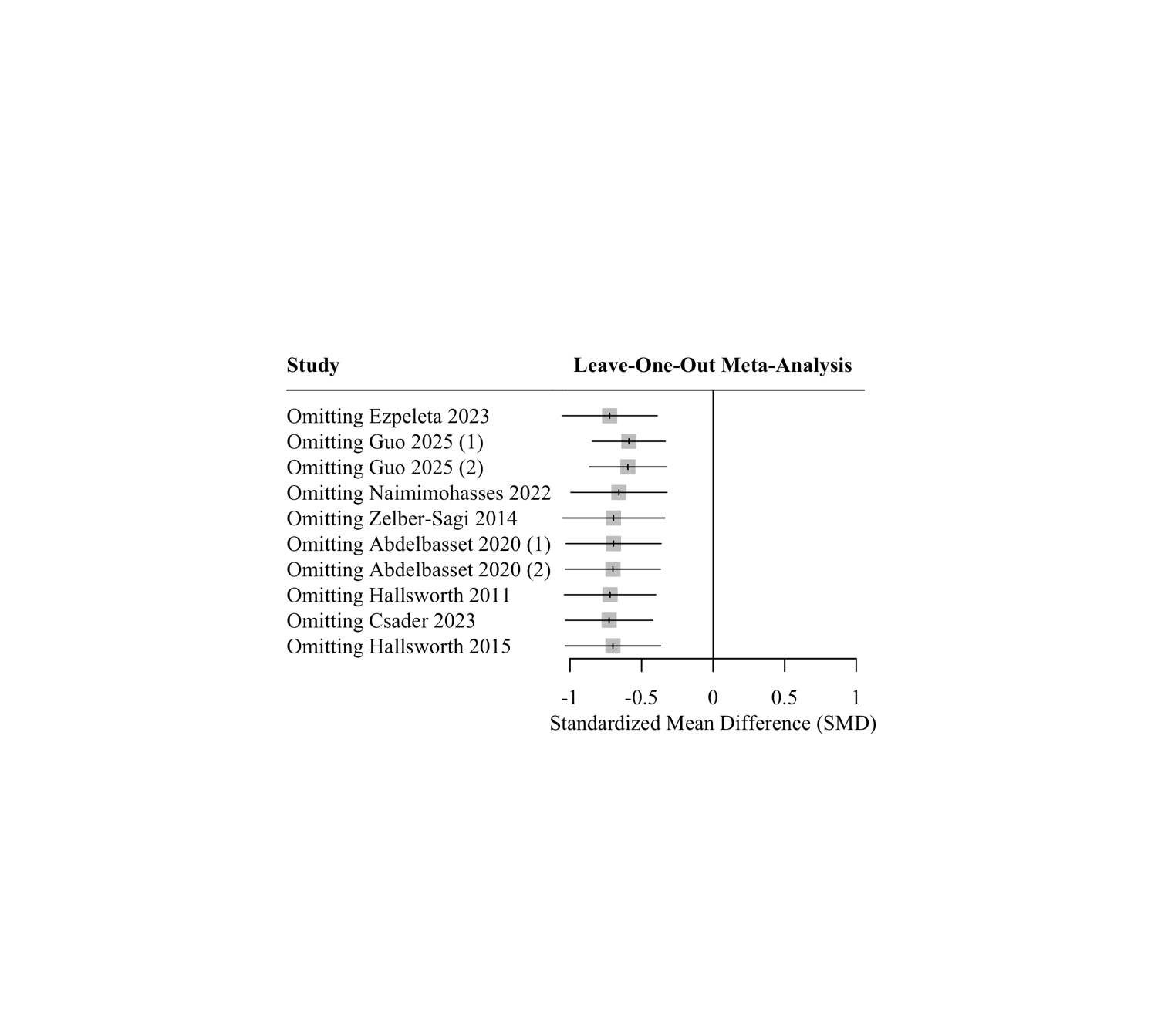


Figure A9 Leave-One-Out Meta-Analysis of Liver fat Levels


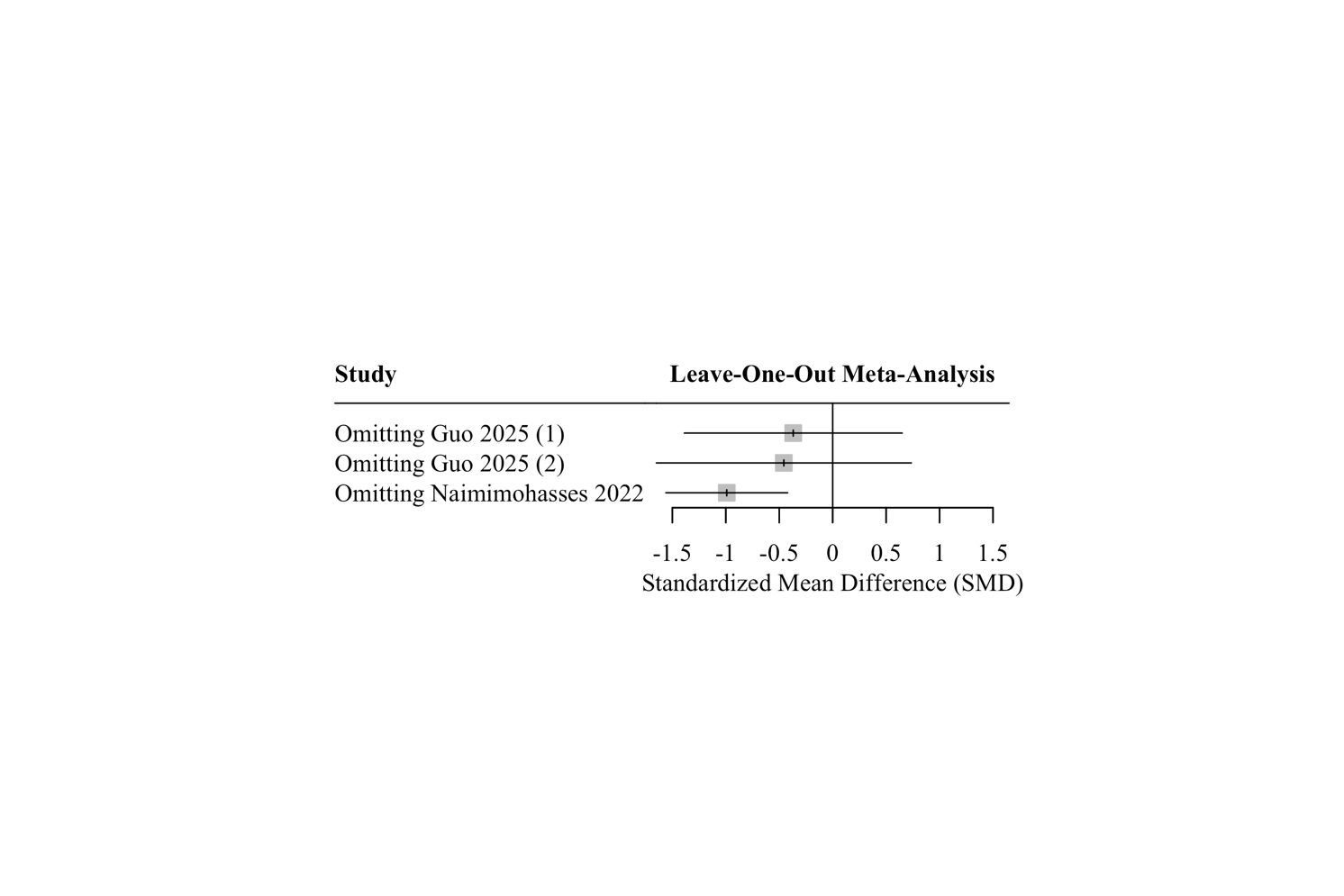


Figure A10 Leave-One-Out Meta-Analysis of Liver stiffness Levels


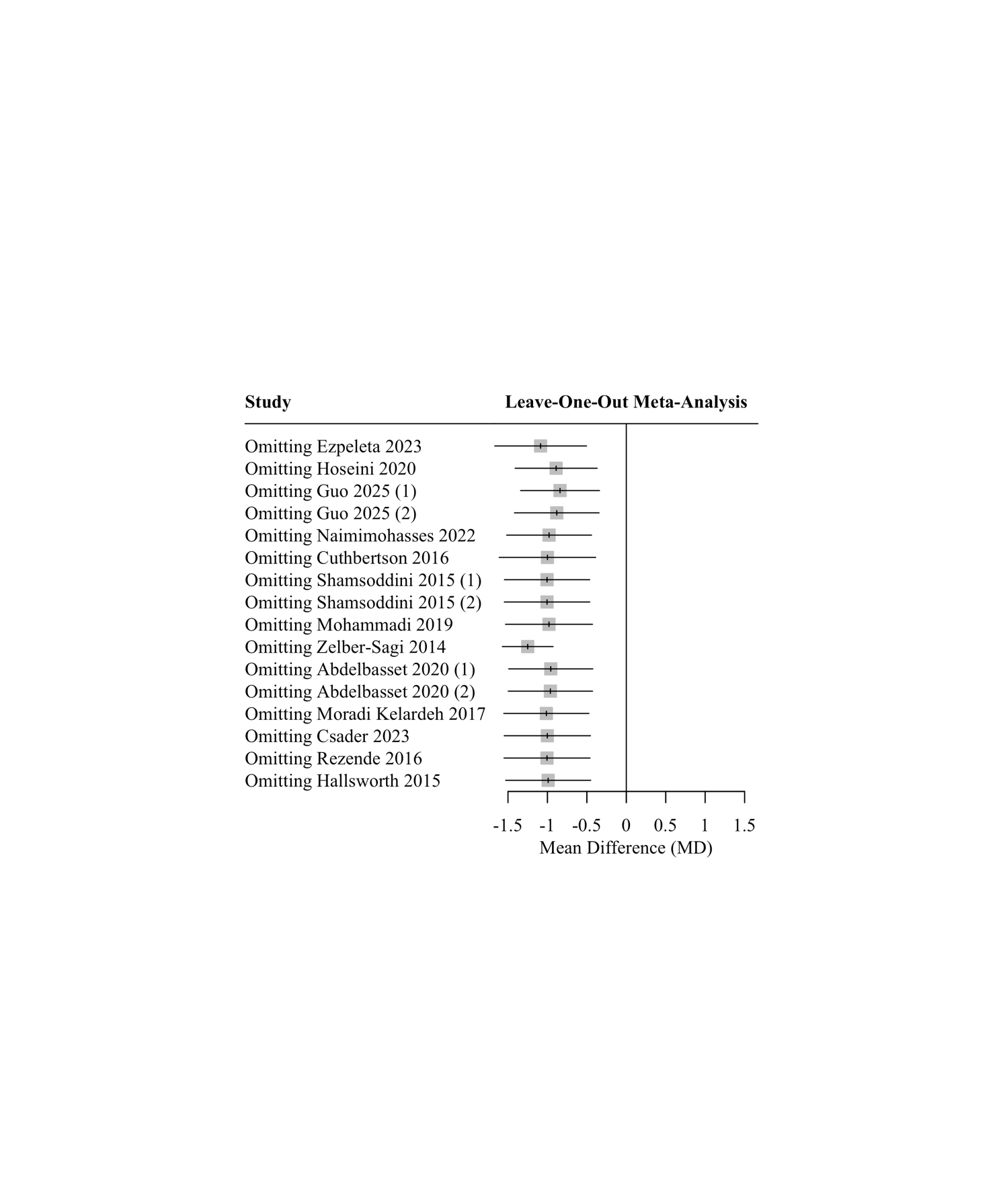


Figure A11 Leave-One-Out Meta-Analysis of BMI Levels


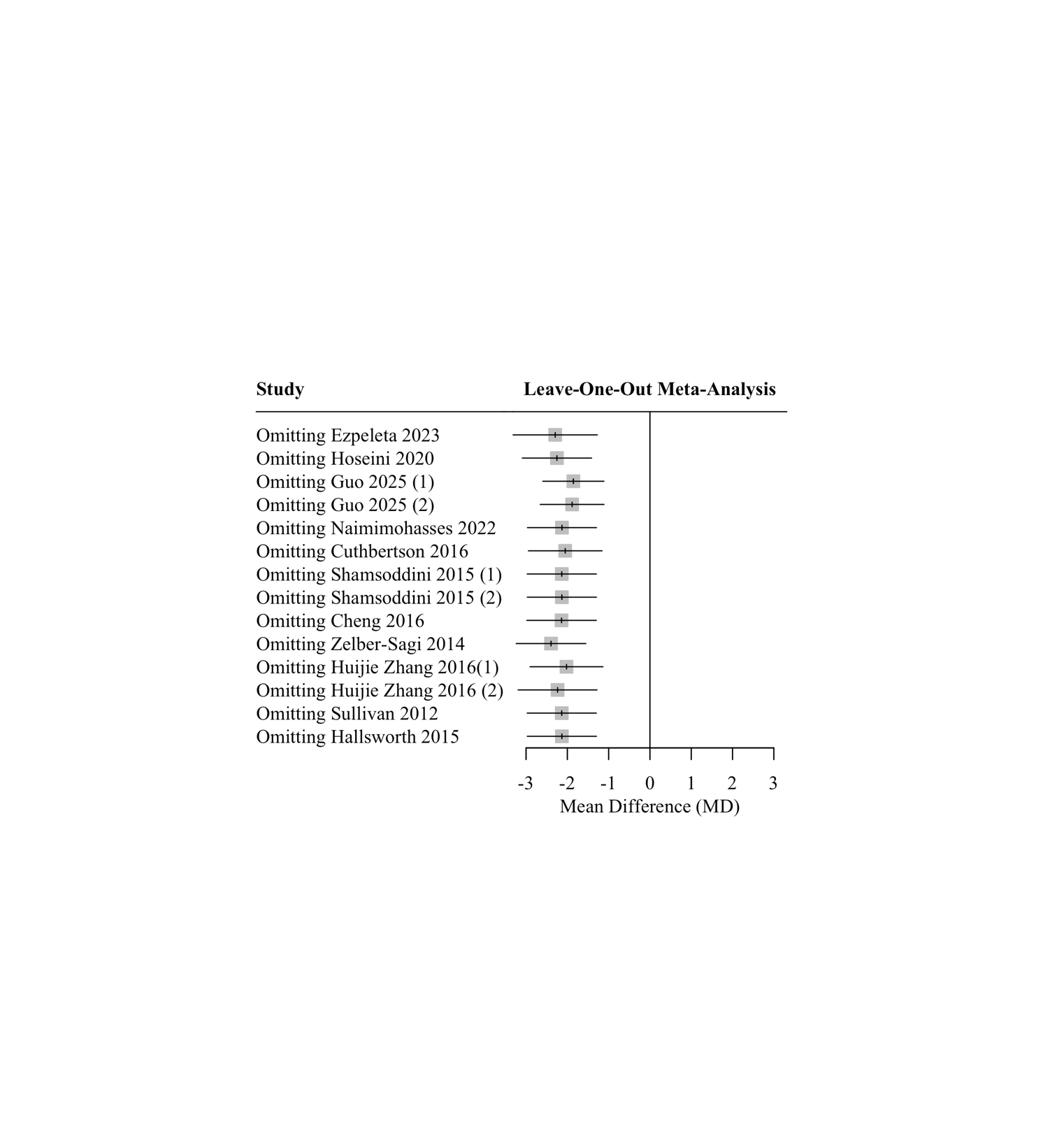


Figure A12 Leave-One-Out Meta-Analysis of weight Levels


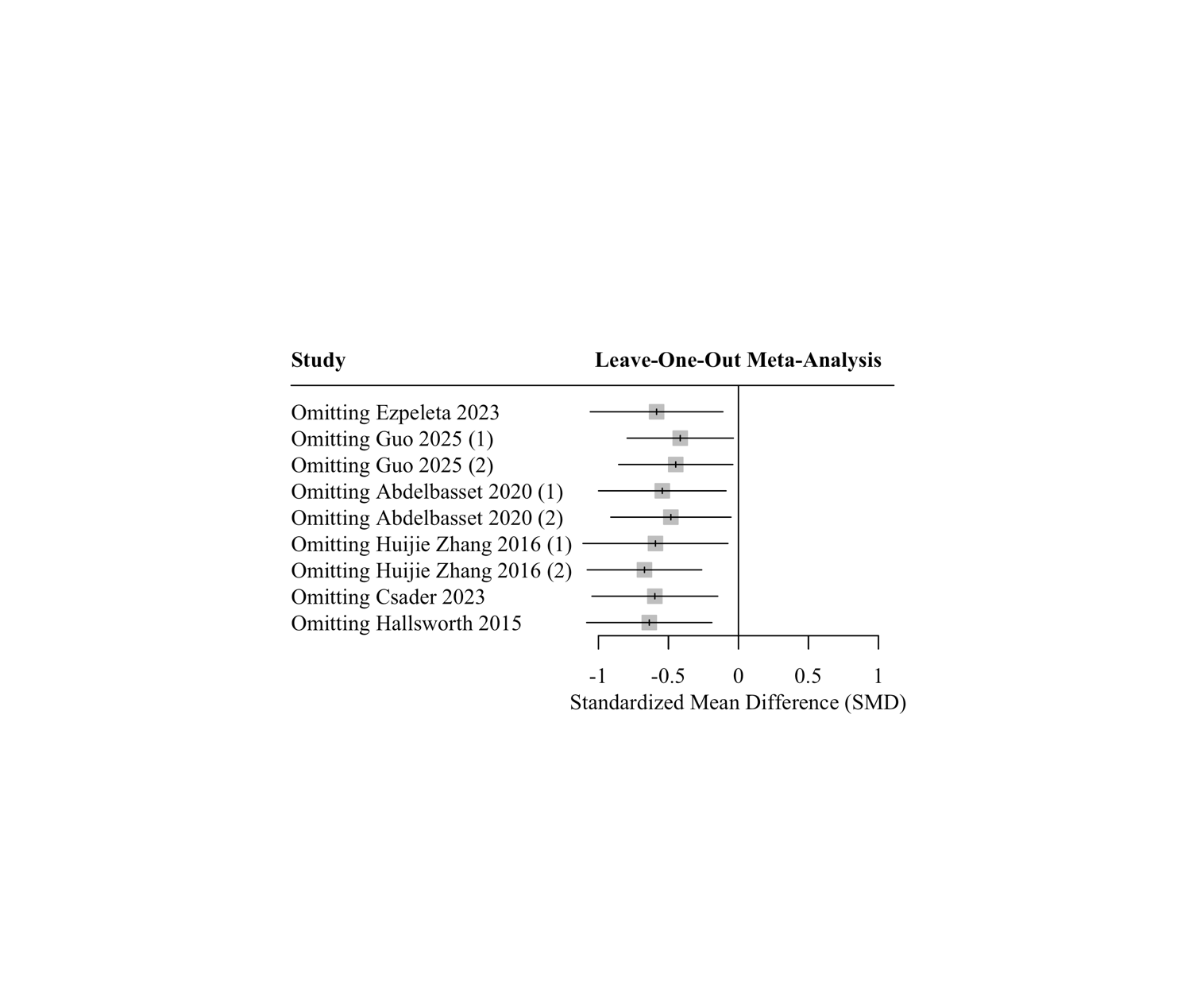


Figure A13 Leave-One-Out Meta-Analysis of Visceral Adipose Tissue Levels


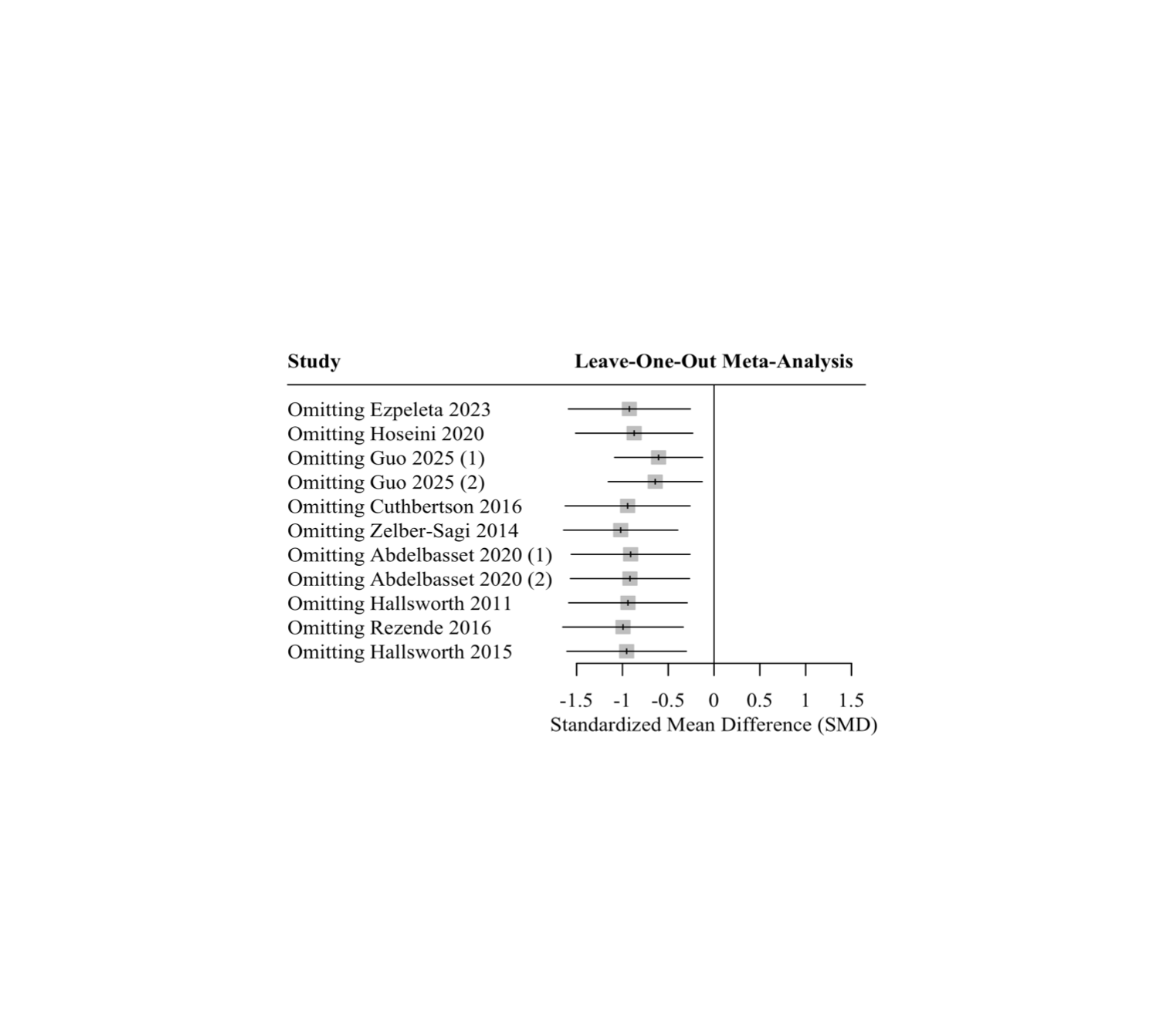


Figure A14 Leave-One-Out Meta-Analysis of Homeostasis Model Assessment of Insulin Resistance Levels


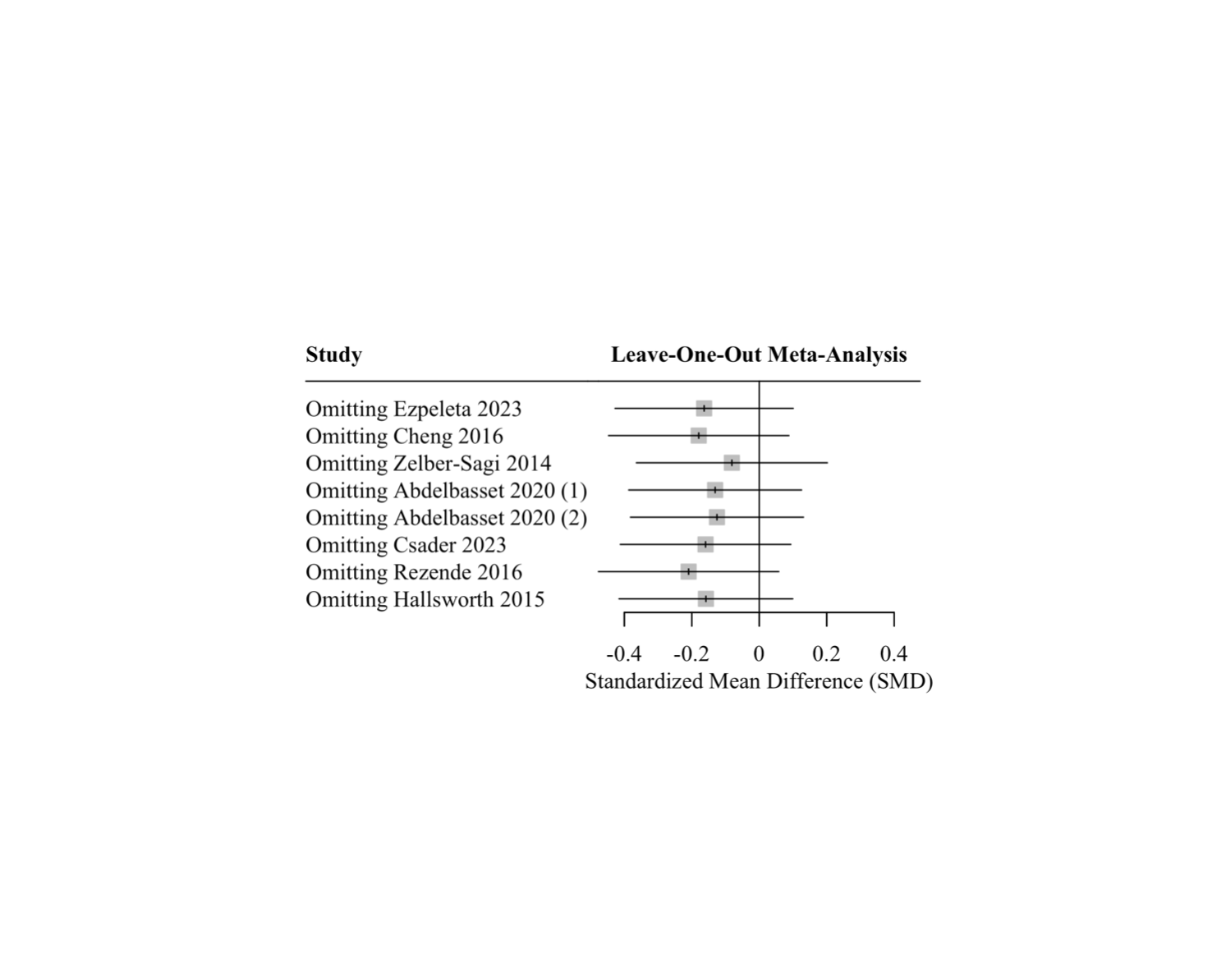


Figure A15 Leave-One-Out Meta-Analysis of Glycated Hemoglobin Percentage Levels


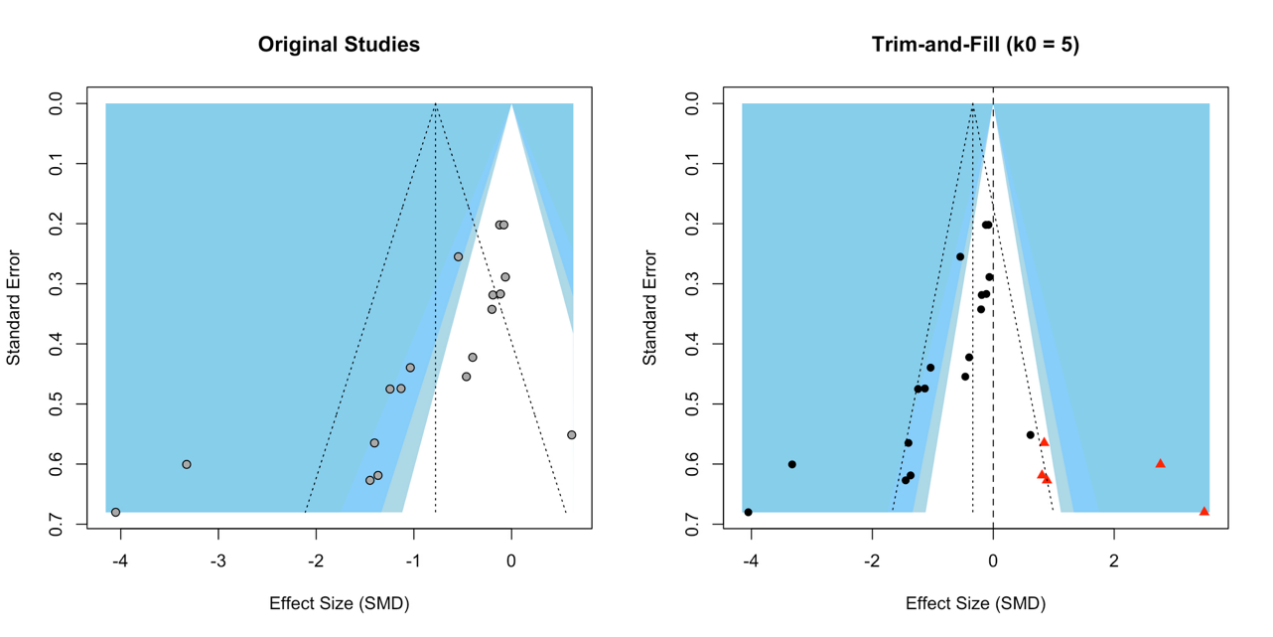


Figure A16 Trim-and-Fill Analysis of Alanine Aminotransferase Levels to Address Publication Bias


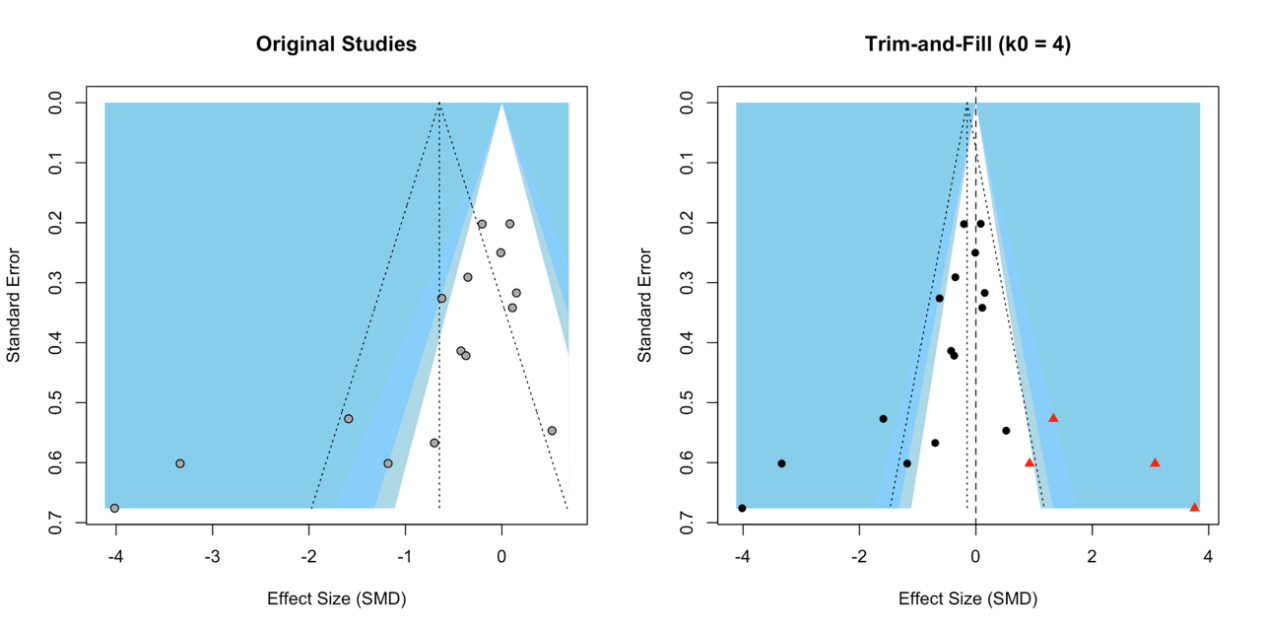


Figure A17 Trim-and-Fill Analysis of Aspartate Aminotransferase Levels to Address Publication Bias


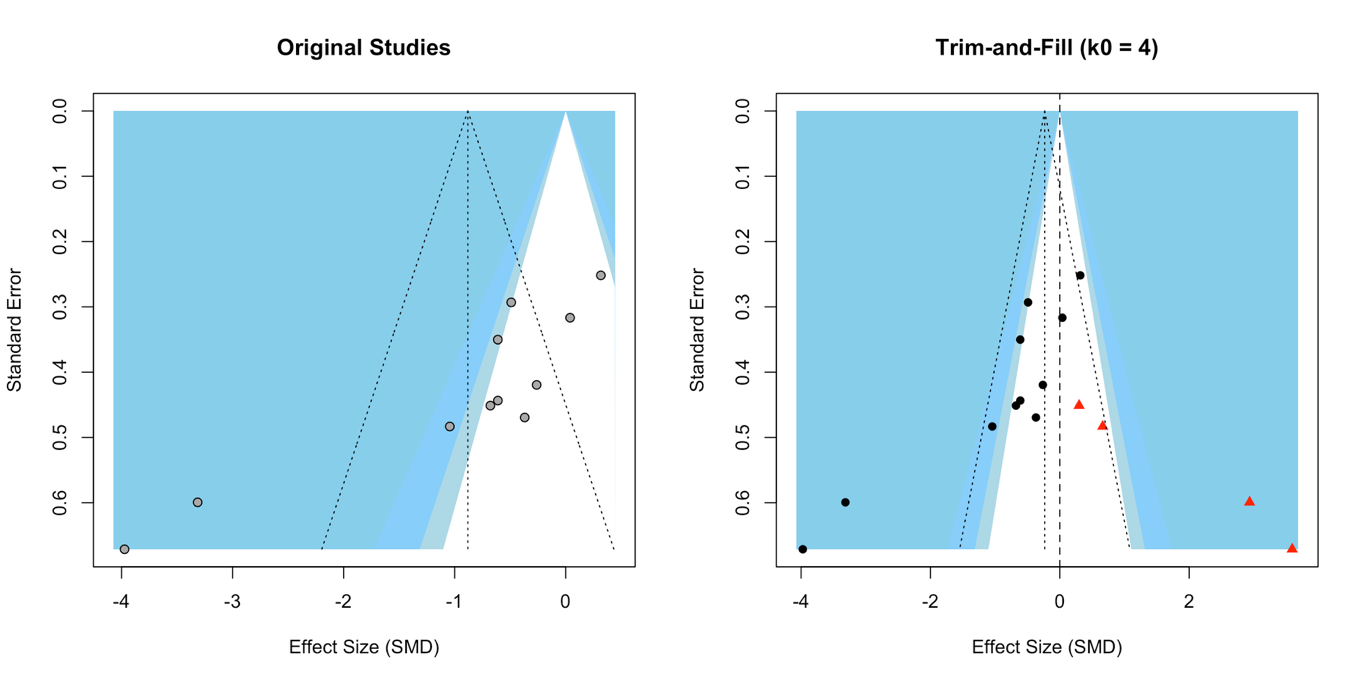


Figure A18 Trim-and-Fill Analysis of Homeostasis Model Assessment of Insulin Resistance Levels to Address Publication Bias


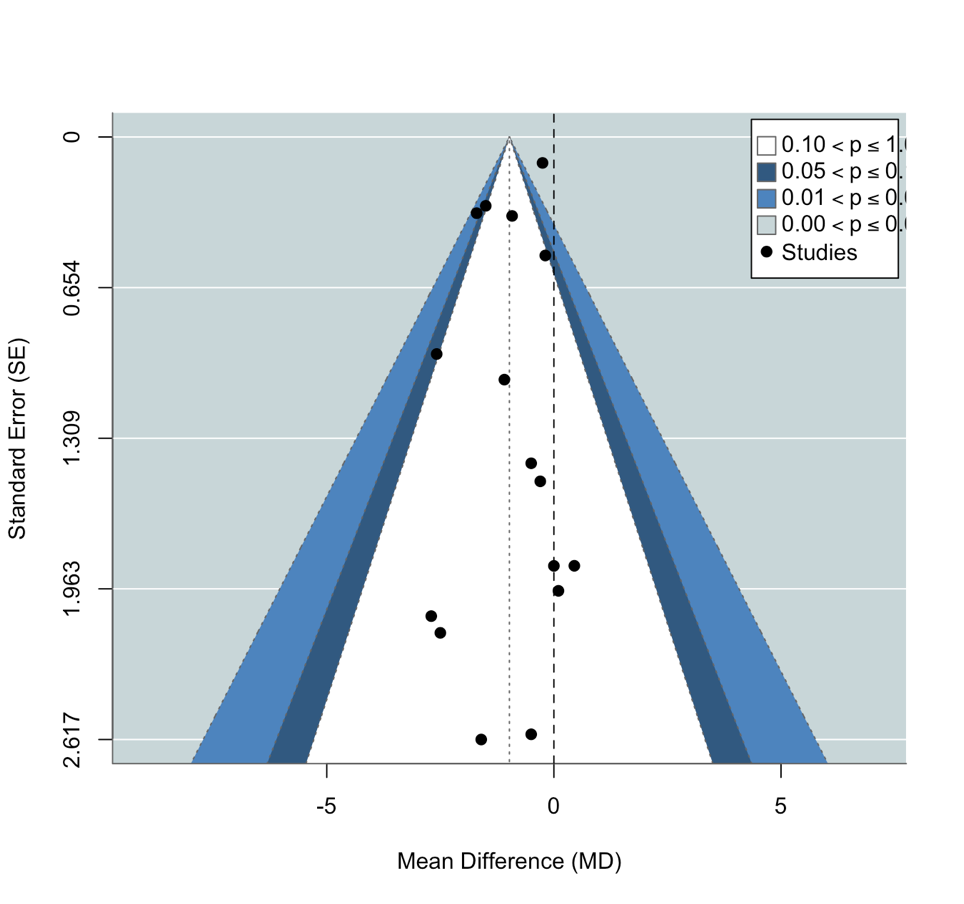


Figure A19 Funnel Plot of the Effect of Exercise on BMI


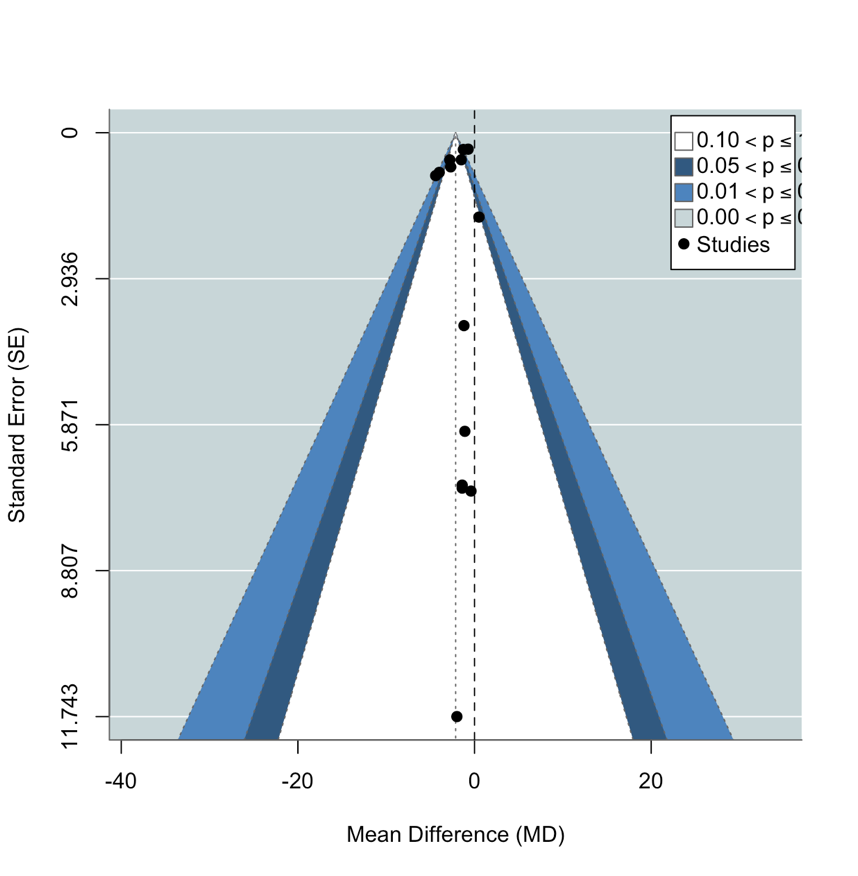


Figure A20 Funnel Plot of the Effect of Exercise on weight


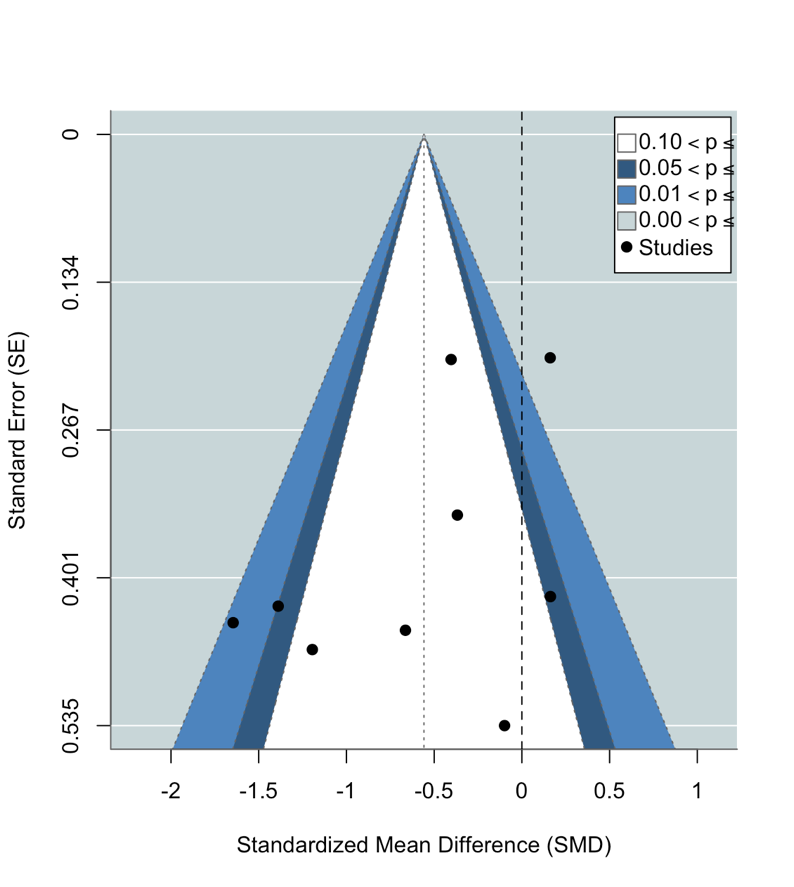


Figure A21 Funnel Plot of the Effect of Exercise on Visceral Adipose Tissue


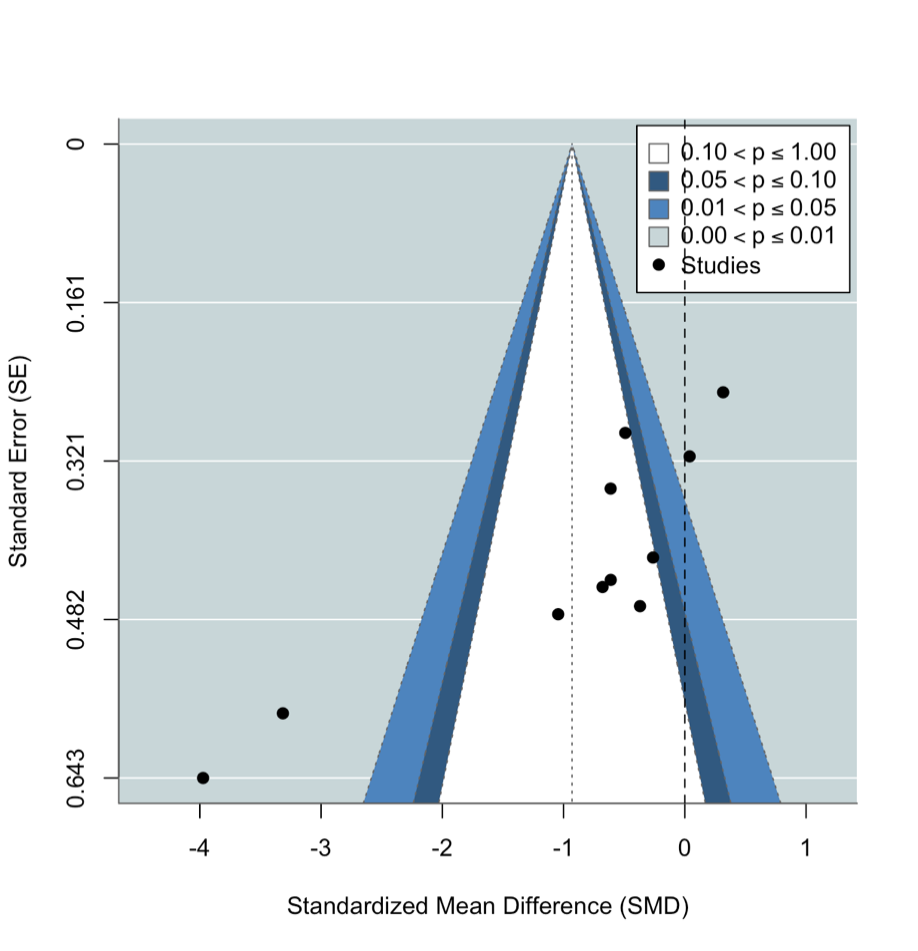


Figure A22 Funnel Plot of the Effect of Exercise on Homeostasis Model Assessment of Insulin Resistance


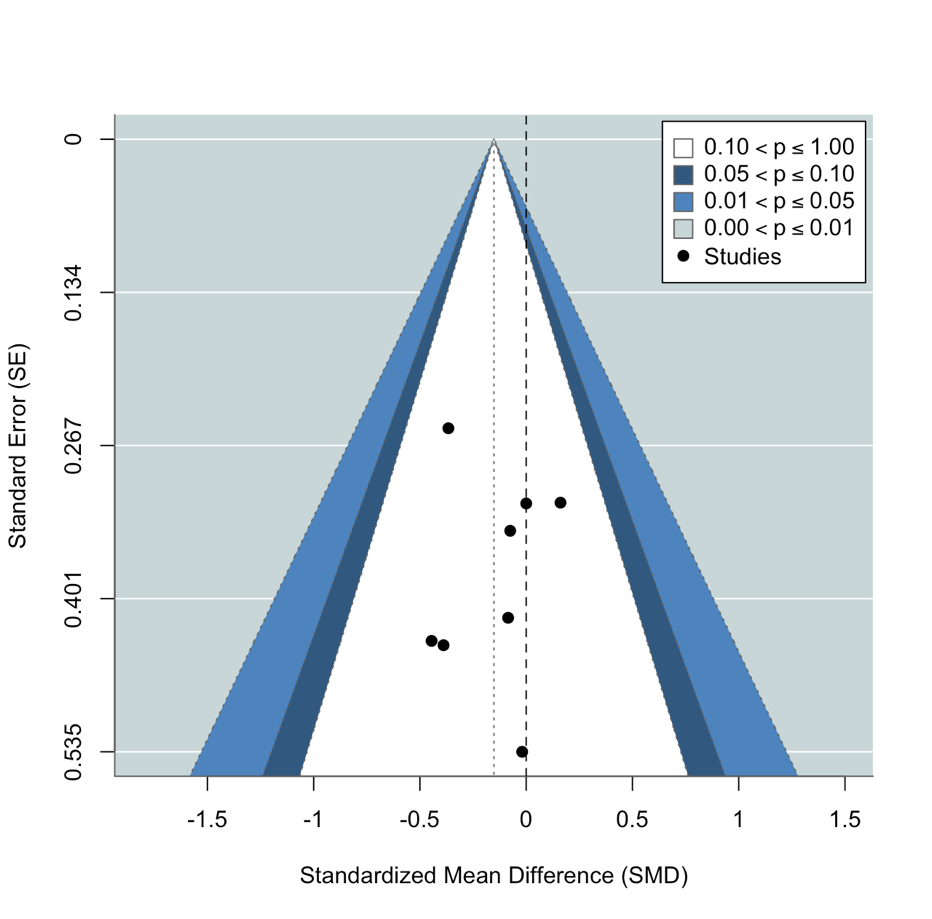


Figure A23 Funnel Plot of the Effect of Exercise on Glycated Hemoglobin Percentage

**
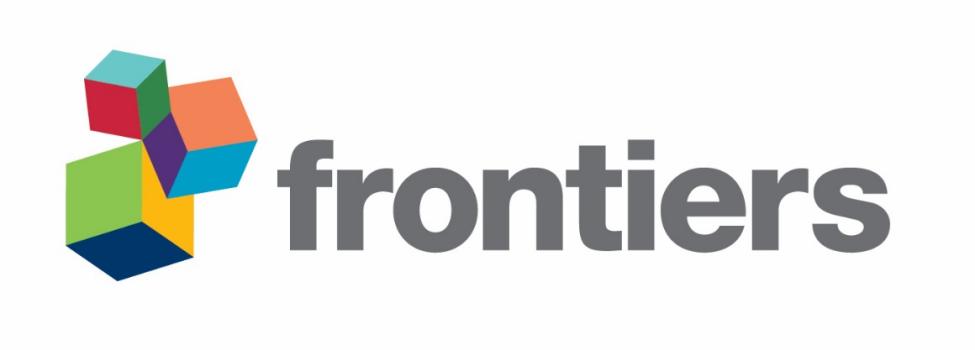
**
